# *Hormad1* Mutation Disrupts Synaptonemal Complex Formation, Recombination, and Chromosome Segregation in Mammalian Meiosis

**DOI:** 10.1371/journal.pgen.1001190

**Published:** 2010-11-04

**Authors:** Yong-Hyun Shin, Youngsok Choi, Serpil Uckac Erdin, Svetlana A. Yatsenko, Malgorzata Kloc, Fang Yang, P. Jeremy Wang, Marvin L. Meistrich, Aleksandar Rajkovic

**Affiliations:** 1 Department of Obstetrics and Gynecology, Baylor College of Medicine, Houston, Texas, United States of America; 2 Department of Obstetrics and Gynecology and Reproductive Sciences, Magee Women's Research Institute, University of Pittsburgh, Pittsburgh, Pennsylvania, United States of America; 3 Fertility Center of CHA General Hospital, CHA Research Institute, CHA University, Seoul, Korea; 4 Department of Molecular and Human Genetics, Baylor College of Medicine, Houston, Texas, United States of America; 5 Department of Surgery, The Methodist Hospital and The Methodist Hospital Research Institute, Houston, Texas, United States of America; 6 Department of Animal Biology, University of Pennsylvania, Philadelphia, Pennsylvania, United States of America; 7 Department of Experimental Radiation Oncology, The University of Texas M. D. Anderson Cancer Center, Houston, Texas, United States of America; Stowers Institute for Medical Research, United States of America

## Abstract

Meiosis is unique to germ cells and essential for reproduction. During the first meiotic division, homologous chromosomes pair, recombine, and form chiasmata. The homologues connect via axial elements and numerous transverse filaments to form the synaptonemal complex. The synaptonemal complex is a critical component for chromosome pairing, segregation, and recombination. We previously identified a novel germ cell–specific HORMA domain encoding gene, *Hormad1*, a member of the synaptonemal complex and a mammalian counterpart to the yeast meiotic HORMA domain protein Hop1. *Hormad1* is essential for mammalian gametogenesis as knockout male and female mice are infertile. *Hormad1* deficient (*Hormad1^−/^* ^−^) testes exhibit meiotic arrest in the early pachytene stage, and synaptonemal complexes cannot be visualized by electron microscopy. *Hormad1* deficiency does not affect localization of other synaptonemal complex proteins, SYCP2 and SYCP3, but disrupts homologous chromosome pairing. Double stranded break formation and early recombination events are disrupted in *Hormad1^−/^* ^−^ testes and ovaries as shown by the drastic decrease in the γH2AX, DMC1, RAD51, and RPA foci. HORMAD1 co-localizes with γH2AX to the sex body during pachytene. BRCA1, ATR, and γH2AX co-localize to the sex body and participate in meiotic sex chromosome inactivation and transcriptional silencing. *Hormad1* deficiency abolishes γH2AX, ATR, and BRCA1 localization to the sex chromosomes and causes transcriptional de-repression on the X chromosome. Unlike testes, *Hormad1^−/^* ^−^ ovaries have seemingly normal ovarian folliculogenesis after puberty. However, embryos generated from *Hormad1^−/^* ^−^ oocytes are hyper- and hypodiploid at the 2 cell and 8 cell stage, and they arrest at the blastocyst stage. HORMAD1 is therefore a critical component of the synaptonemal complex that affects synapsis, recombination, and meiotic sex chromosome inactivation and transcriptional silencing.

## Author Summary

The biology of germ cells is intimately intertwined with meiosis. Meiosis I is a unique biological event, when chromosomes pair, recombine, and segregate. The synaptonemal complex is a protein lattice that enables chromosome pairing and recombination and is unique to meiosis I. Meiosis I requires a subset of factors that are unique to germ cells and meiosis. Germ cell–specific factors are known to play crucial roles during formation of the synaptonemal complex and include synaptonemal complex proteins SYCP1, SYCP2, and SYCP3, among others. We discovered a mouse HORMA domain containing protein, *Hormad1* (*Nohma*), which is germ cell–specific and essential for male and female fertility. Mice deficient in *Hormad1* have severe defects in early recombination, synapsis, and segregation—functions attributed to yeast HORMA domain containing protein, Hop1. Moreover, *Hormad1* is likely a germ cell–specific component of the meiotic sex chromosome inactivation and transcriptional silencing complex.

## Introduction

Mammalian meiosis is unique to germ cells and a critical step in sexual reproduction. Meiosis reduces the chromosome complement to haploidy in preparation for fertilization. The first meiotic division is unique in pairing of homologous chromosomes, homologous recombination, and formation of chiasmata. The reduction in chromosome numbers happens when homologous chromosomes segregate to opposite poles during the first meiotic division. Proper disjunction (separation) requires crossovers (manifested cytologically as chiasmata). The sister chromatids organize along structures called axial elements (AEs) and transverse elements connect AEs to form the synaptonemal complex (SC) [1]. SC is a proteinaceous structure that connects paired homologous chromosomes during prophase I of meiosis, and SC is critical for wild-type levels of crossovers to occur during meiosis. AEs are critical part of the SCs and mutations in proteins that form AEs disrupt sister chromatid cohesion, recombination, and chromosome segregation [2]–[4]. Proteins with HORMA domain are critical components of the axial elements [5]. HORMA domain proteins are predicted to form globular structure that may sense specialized chromatin states, such as those associated with double strand breaks (DSBs) or other forms of DNA damage [5]. Several mammalian proteins that contain HORMA domain, such as mitotic arrest deficient protein 2, MAD2, are essential for mitosis [6]–[7]. Mice lacking MAD2 unsurprisingly die during early embryogenesis [7]. In lower organisms, several meiotic specific HORMA proteins are known and all are critical for meiosis. These HORMA proteins are: Hop1 [8] and Red1 [9] in yeast; Him-3 [10] in nematodes; and Asy1 [11] in plants. Him-3 localizes to the axial cores of both synapsed and unsynapsed chromosomes. *C. elegans* Him*-3* mutants are deficient in chromosome pairing, synapsis, and the regulation of double strand break repair [10], [12]–[13]. Synapsis in both male and female *Asy1* mutants is disrupted [14]–[15]. In yeast, plants, and nematodes, HORMA domain proteins are critical components of the synaptonemal complex and essential for meiosis I. Others and we identified a previously uncharacterized gene that we named *Nohma*, later re-named to *Hormad1* [16]–[18]. *Hormad1* encodes a protein that contains a HORMA domain, and unlike *Mad2*, *Hormad1* expression is germ cell–specific [16]. Mouse and human HORMAD1 are highly conserved and share 77% amino acid identity overall, and share 89% amino acid identity in the HORMA domain. Moreover, mouse and human HORMAD1 HORMA domains share 28% amino acid identity with Hop1 HORMA domain. Hop1 in yeast appears to bind near or at the sites of DSB formation and may modulate the initial DSB cleavage [19]. *Hop1* mutants in yeast have reduced number of DSBs [20], and Hop1 may participate in recruiting DMC1, RAD51 and other proteins that are required for DNA repair during meiotic synapsis and recombination [19]–[20]. Phosphorylation of Hop1 by Mec1/Tel1 yeast kinases is important for interhomologue recombination and prevents DMC1-independent repair of meiotic DSBs [21]. Here we report that HORMAD1 is likely the mammalian counterpart of Hop1, and that HORMAD1 deficiency disrupts mammalian synaptonemal complex formation, meiotic recombination, and chromosome segregation.

## Results

### HORMAD1 is essential for spermatogenesis

We previously showed that *Hormad1* RNA expression in testes began at postnatal day 10, with little expression detected at birth or postnatal day 5 [16]. *Hormad1* RNA expression pattern coincided with the onset of meiosis, and appearance of primary spermatocytes in the developing testes. *In situ* hybridization with anti-sense *Hormad1* riboprobe revealed that *Hormad1* expression was confined to germ cells, and specifically spermatocytes, with no signal detected in spermatogonia or sertoli cells [16]. We generated antibodies against HORMAD1 and studied its protein localization pattern in testes. HORMAD1 localized exclusively in germ cells, specifically in zygotene, and early pachytene spermatocytes as previously described for the RNA expression [16].

Since HORMAD1 protein showed localization consistent with its potential role in meiosis I and contains the HORMA domain, we disrupted the *Hormad1* gene to examine its requirement for germ cell development and meiosis in mouse. *Hormad1* is located on chromosome 3 and composed of sixteen exons. We deleted exons 4 and 5 (Figure S1A), and this mutation is predicted to remove 33 amino acids from the highly conserved HORMA domain and to cause a frame shift mutation. Small amounts of truncated *Hormad1* RNA transcripts were detectable on RT-PCR, and Western blots on testes extracts showed absence of HORMAD1 protein in knockout mice as expected (Figure S1B and S1C).

Female and male heterozygote matings produced expected Mendelian ratios, averaged 8.1±2 pups per litter (*n* = 20 breeding pairs) over a 6-month period, and remained fertile for at least 9 months. The litter size was statistically not significantly different from the wild-type average (8.4±2 pups per litter). Male and female mice heterozygous for the mutation (*Hormad1^+/−^*) were fertile with grossly normal male and female gonadal morphology and histology. However, both *Hormad1^−/−^* males and females were infertile with no pups produced over a period of 6 months from mating with wild-type female and male mice, respectively.

While ovaries showed no gross morphologic differences between the knockout and wild-type mice, *Hormad1* knockout adult testes were significantly smaller than the wild-type testes (Figure S2A). Testes in the 7-day-old *Hormad1^−/−^* mice were grossly normal and weighed 8.0±2.0 mg/pair and did not significantly differ from the wild-type, 9.0±3.0 mg/pair of testes. By 4 weeks of postnatal life, the knockout testes (47±6 mg/pair) were 50% of the wild-type weight (94±1.7 mg/pair), and by 8 weeks the knockout testes (60±7.7 mg/pair) were 27% of the wild-type weight (225±2.7 mg/pair) (Figure S2B).

**Figure 1:**
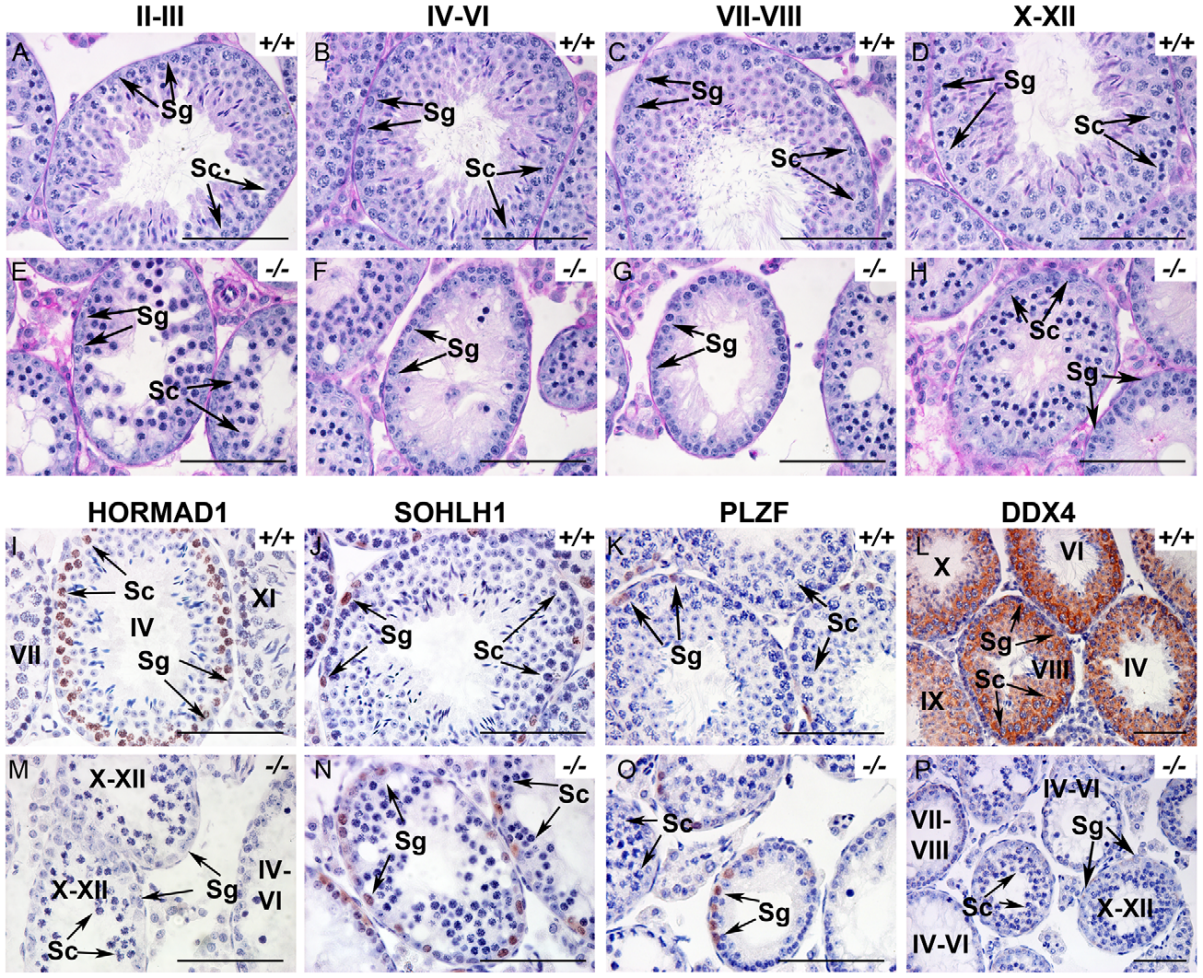
HORMAD1 is required for spermatogenesis. (A–H). Periodic acid/Schiff reagent (PAS) stained cross sections from 6 weeks old wild-type testes (A-D) and *Hormad1 ^−/−^* (E-H) testes. Seminiferous tubule stages are shown above the panels. Lack of mature sperm and arrest in spermatogenesis is shown at different tubular stages. (I-P). Immunohistochemistry (IHC) of 6 weeks old wild type and *Hormad1^−/−^* testes with anti HORMAD1, SOHLH1, PLZF and DDX4 antibody. IHC with anti-HORMAD1 antibody shows HORMAD1 expression (brown) in wild-type stage IV pachytene, and stage XI zygotene spermatocytes (I), while no expression was observed in the knockout (M). SOHLH1 is expressed in differentiating spermatogonia and is present in wild-type and *Hormad1^−/−^* testes as shown by arrows (J and N). PLZF identifies differentiating and self-renewing spermatogonia and is expressed in both wild-type and *Hormad1^−/−^* testes as shown by arrows (K and O). DDX4 is an RNA binding protein specific for germ cells, and is under-expressed in *Hormad1^−/−^* spermatocytes (L and P). Arrows point to Spermatogonia (Sg) and Spermatocytes 1(Sc). Scale bars: 50 µm.

Histology at 6 weeks showed hypocellular seminiferous tubules with clumps of sertoli cells in the lumen. We observed spermatogonia and early spermatocytes, but no post-meiotic germ cells such as spermatids or spermatozoa (Figure 1A–1H). We therefore carefully examined spermatogenesis in *Hormad1^−/−^* mice. Spermatogenesis is a complex process that involves differentiating and proliferating self-renewing spermatogonia that differentiate into spermatozoa. Type A spermatogonia self-renew and can initiate differentiation into Type B spermatogonia which in turn differentiate into primary spermatocytes. Primary spermatocytes undergo meiosis I to form secondary spermatocytes. Secondary spermatocytes enter meiosis II and divide to produce haploid spermatids. We examined *Hormad1* knockout testes histology during gonadal development to determine the stage at which spermatogenesis is disrupted. Identical testes weights at postnatal day 7, and similar histology between *Hormad1^−/−^* and wild-type testes argue that pre-spermatogonia in *Hormad1^−/−^* testes proliferate into Type A spermatogonia without major disruption. Immunohistochemistry with antibodies directed against PLZF and SOHLH1, markers that identify self-renewing (PLZF) and differentiating spermatogonia (SOHLH1), showed the presence of both proteins in the wild-type as well as the knockout animals, confirming that spermatogonia are unaffected (Figure 1J, 1K, 1N, 1O). At postnatal day 10, testes contain preleptotene/leptotene primary spermatocytes, and there was no gross difference between wild-type and *Hormad1^−/−^* testes. At 14 days, testes contain pachytene spermatocytes, and there was a significant difference between the wild-type and *Hormad1^−/−^* testes, with many apoptotic cells and few pachytene spermatocytes in *Hormad1^−/−^* testes (), and rising apoptotic index with age in the knockout as compared to the wild-type (Figure S3K). We counted leptotene, zygotene and pachytene spermatocytes in 6 week old wild-type and *Hormad1^−/−^* testes. *Hormad1^−/−^* testes showed declining number of spermatocytes beginning in stages II-III with 28±8 spermatocytes as compared to 52±12 in the wild-type (Figure 1A and 1E). No spermatocytes were noted in stages IV-IX in the *Hormad1^−/−^* testes (Figure 1F and 1G), and no significant difference was noted in the spermatocyte number in stages X-XII between the wild-type and knockout testes (Figure 1D and 1H). These results indicate that *Hormad1* deficiency in the male gonad caused meiotic arrest at the pachytene stage.

### HORMAD1 is critical for chromosome synapsis

**Figure 2:**
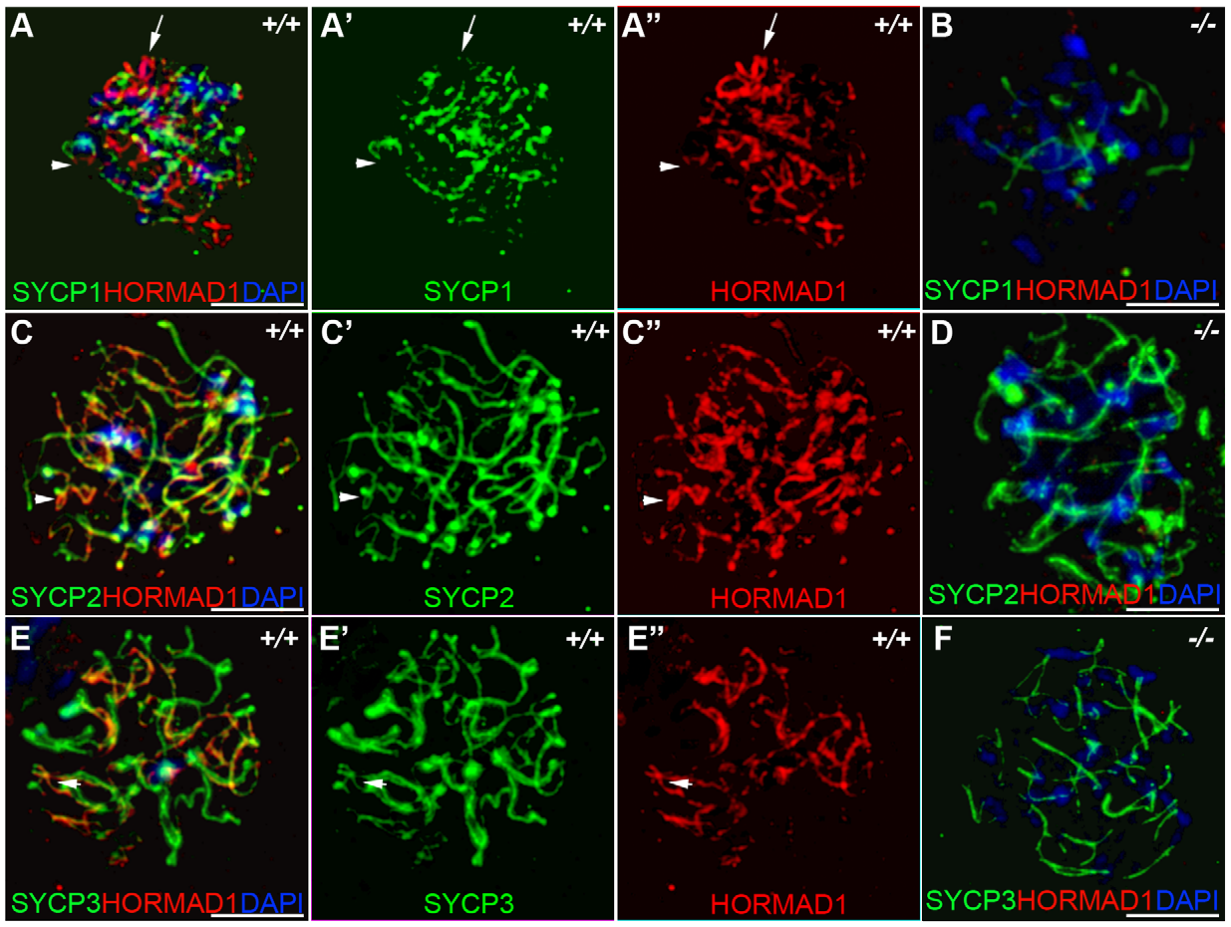
HORMAD1 co-localizes with SYCP2 and SYCP3, but not with SYCP1. Chromosomal spread assay in wild-type (+/+) and *Hormad1^−/−^* (*−*/*−*) zygotene stage spermatocytes. (A–B) Immunofluorescence staining with anti-SYCP1 (Green) and anti-HORMAD1 (Red) antibody. HORMAD1 preferentially localizes to unsynapsed regions of chromosome axes (arrow head) and sex body (arrow) but does not co-localize with SYCP1 (A-A”). *Hormad1* deficiency does not affect SYCP1 localization to the axes (B). HORMAD1 co-localizes with SYCP2 on the unsynapsed chromosome axes (C-C”), but HORMAD1 deficiency does not affect SYCP2 localization to the axes (D). SYCP3, another integral and critical component of the synaptonemal complex co-localizes with HORMAD1 on unsynapsed axes (E-E”). Similar to SYCP1 and SYCP2, SYCP3 localizes to the axes despite HORMAD1 deficiency (F). DNA was stained with DAPI. Scale bars: 10 µm.

**Figure 3:**
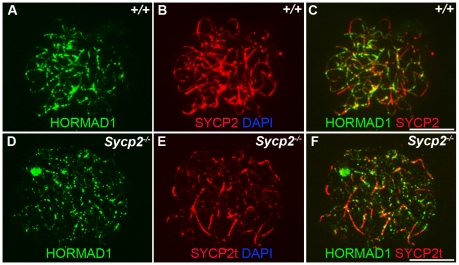
HORMAD1 localization in *Sycp2* mutant spermatocytes. Chromosome spread assay was performed on the wild-type (+/+) (A–C) and *Sycp2^−/−^* (D–F) zygotene spermatocytes with anti-HORMAD1 (green) and anti-SYCP2 (red) antibodies. The truncated SYCP2 protein (SYCP2t) in *Sycp2^−/−^* mice is still made and localizes to axial chromosomal cores[48]. SYCP2t lacks the domain necessary to bind SYCP3. Scale bars: 10 µm.

Previous studies on HORMA domain proteins indicate their specific involvement in cell division. MAD2 is a ubiquitously expressed mammalian HORMA domain protein involved in both meiosis and mitosis [7], while yeast *HOP1*, *RED1*, nematode *HIM3* and plant *ASY1* genes are specifically involved in meiotic segregation, synapsis and recombination [2], [10]–[11], [13], [15], [22]. No mammalian counterparts to Hop1, Red1, Him3 and Asy1 have been functionally evaluated up to date. We previously hypothesized that HORMAD1 is a functional counterpart to Hop1, Him3 and Asy1 [16]. Critical components of the synaptonemal complex include meiosis specific SYCP1, SYCP2 and SYCP3 proteins. SYCP1 is a major component of the transverse filaments, while both SYCP2 and SYCP3 are components of the axial lateral elements [23]–[26]. To determine HORMAD1 localization during meiosis, and whether HORMAD1 localizes to the axial elements, or transverse filaments, we used antibodies against SYCP1, SYCP2 and SYCP3 to study their respective co-localization with HORMAD1. HORMAD1 co-localized with SYCP3 and SYCP2 but did not co-localize with SYCP1, which indicates that HORMAD1 is located along the axial elements (Figure 2A, 2C, and 2E). Recent studies also show that HORMAD1 localizes to the axial elements [17]–[18]. We also studied whether absence of SYCP2 affected HORMAD1 localization along the chromosomes. HORMAD1 localization is independent of major germ cell–specific components of the axial elements of the synaptonemal complex because neither *Sycp2* (Figure 3A–3F) nor *Sycp3* mutation affected HORMAD1 localization to the axial elements [18].

We examined localization of germ cell–specific synaptonemal complex proteins SYCP1, SYCP2 and SYCP3 in *Hormad1^−/−^* testes. SYCP1 is a major component of the transverse filaments and is known to form fibrillar structures in *Sycp3* mutant spermatocytes [27]. However, the fibers are truncated, contain axial gaps, and do not associate with the centromeres of the meiotic chromosomes. We also observed truncated fibers with anti-SYCP1 antibodies in *Hormad1^−/−^* mice (Figure 2B). HORMAD1 is therefore not necessary for SYCP1 binding to the chromatin. We also examined localization of SYCP2 and SYCP3 proteins in *Hormad1^−/−^* mice. The localization of SYCP2 and SYCP3 to the chromatin was not significantly affected by the lack of HORMAD1 (Figure 2D and 2F). These results indicate that HORMAD1 is not necessary for SYCP2 and SYCP3 localization to the axial elements.

**Figure 4:**
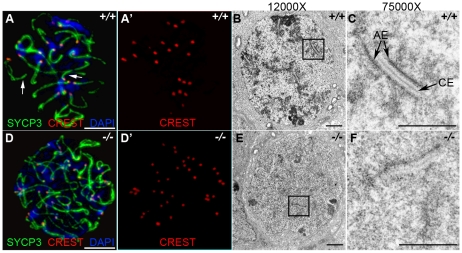
HORMAD1 is required for chromosome synapsis during male meiosis. (A and D) Immunofluorescence with CREST (red) and anti-SYCP3 (green) antibodies. Anti-CREST antibody recognizes chromosome centromeres. Synapsed wild-type zygotene spermatocytes contain about 20 CREST foci (n = 50) (A-A'). However, *Hormad1^−/−^* spermatocytes contain approximately 40 CREST foci (n = 50), an indication that synapsis is disrupted (D-D'). Arrow indicates chromosome synapses in the wild-type. Scale bars: 10 µm. (B–C, E–F) Electron microscopy analysis of synaptonemal complexes in 2 week old wild-type and *Hormad1^−/−^* spermatocyte at different magnifications. The typical, tripartite structure of the synaptonemal complex consists of one central element (CE) that connects with two axial elements (AE) (C). We did not identify normal tripartite synaptonemal complex structure in *Hormad1^−/−^* spermatocytes. Scale bars: 1 µm.

The deficiency in synaptonemal complex proteins such as SYCP3 is known to affect chromosome synapsis [27]. In order to determine the effect of HORMAD1 deficiency on chromosome synapsis during meiosis I, we utilized CREST sera. CREST sera labels centromeres and allows the determination of the pairing status during meiosis [28]. In the wild type spermatocytes, prior to the synapsis, 40 centromeres are usually observed in the leptotene stage. The number of visible centromeres become reduced as the synapsis of homologues progresses. At the completion of the synapsis in pachytene, 20 centromeric foci are usually observed corresponding to 19 autosomal homologues and partially paired X-Y chromosomes. We examined CREST foci formation in *Hormad1^−/−^* spermatocytes. Examination of over 100 *Hormad1^−/−^* spermatocytes and oocytes in meiosis I, revealed greater than 20 centromeric foci in both male and female germ cells, most containing 40 CREST foci (Figure 4A, 4D, and data not shown). These results indicate that *Hormad1* deficient germ cells cannot complete homologous chromosome pairing, and *Hormad1* is therefore critical for chromosome synapsis during meiosis.

Our experimental evidence strongly suggests that HORMAD1 localizes to the axial core and is yet another critical component of the synaptonemal complex. To determine the effect of *Hormad1* deficiency on the structure of the synaptonemal complex, we visualized synaptonemal complexes during meiosis I in wild-type and *Hormad1^−/−^* spermatocytes using electron microscopy. In the wild-type, synaptonemal complexes were well visualized during the pachytene stage of meiosis (Figure 4B and 4C). Electron microscopy examination of one hundred and ten *Hormad1^−/−^* spermatocytes from three independent experiments revealed the lack of the typical tripartite synaptonemal complex structure (Figure 4E and 4F). Persistence of pre-synaptic number of centromeric foci (CREST staining) in *Hormad1^−/−^* spermatocytes, as well as non-visualization of the tripartite synaptonemal complex structure by electron microscopy, demonstrate that HORMAD1 is essential for chromosomal synapsis.

### *Hormad1* deficiency disrupts localization of proteins important in early recombination

**Figure 5:**
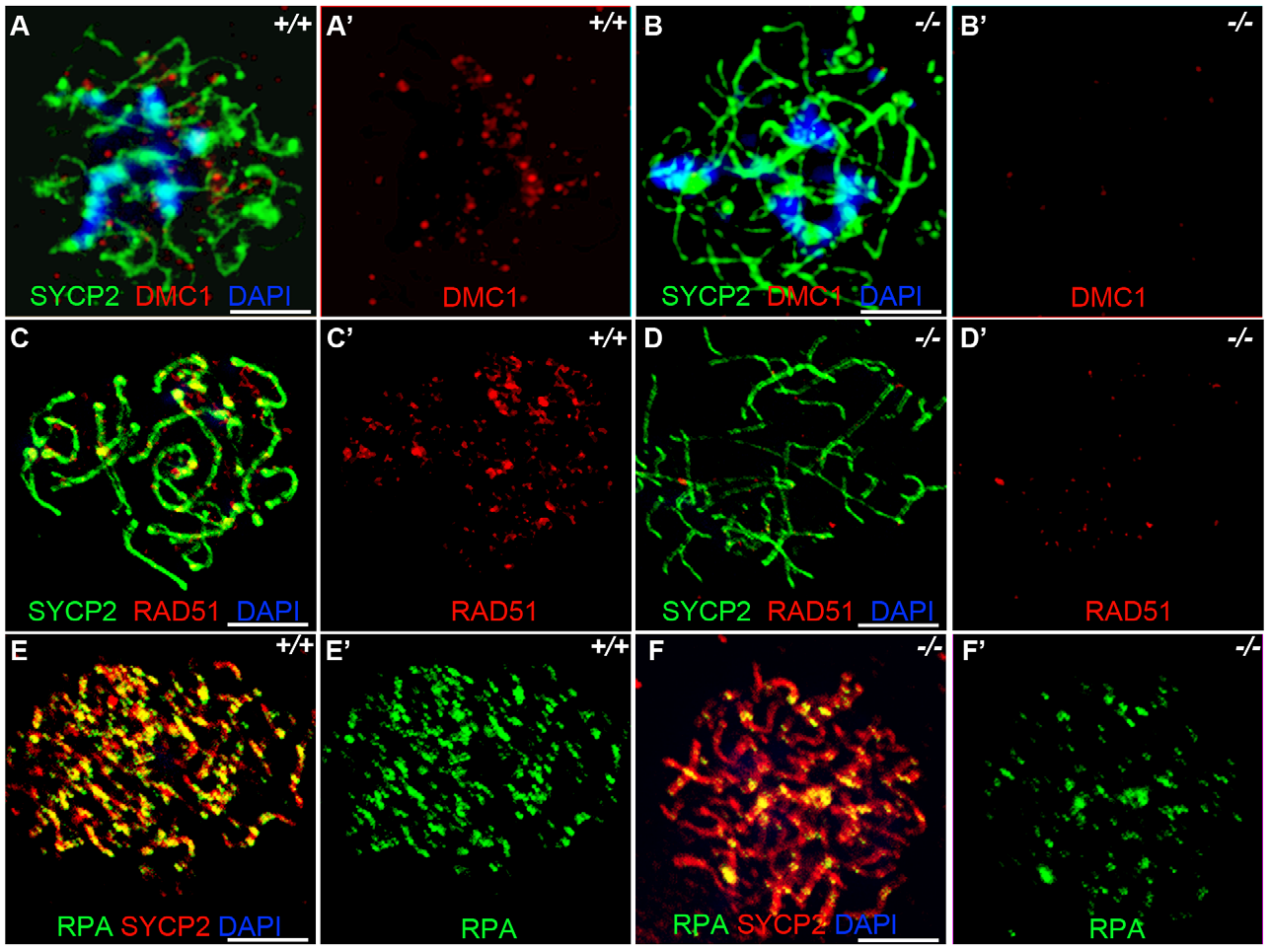
*Hormad ^−/−^* spermatocytes are defective in early recombination. (A-B') Immunofluorescence assay with anti-SYCP2 (Green) and anti-DMC1 (Red) antibody in zygotene spermatocytes. (C-D') Immunofluorescence assay with anti-RAD51 (Red) and anti-SYCP2 (Green) antibody in zygotene spermatocytes. (E-F') Immunofluorescence assay with anti-RPA (Green) and anti-SYCP2 (Red) antibody in zygotene spermatocytes. DMC1 catalyzes DNA strand exchange during recombination, and DMC1, RAD51 and RPA foci mark early recombination events. DMC1, RAD51 and RPA foci are drastically decreased in *Hormad1^−/−^* spermatocytes. Scale bars: 10 µm.

Previous studies have indicated that *Sycp3* deficiency has subtle effects on meiotic recombination [27]. Early recombination events do not seem to be disrupted in *Sycp3*, as similar number of DMC1 foci are present in *Sycp3* mutant and wild-type meiosis [29]. DMC1 is a meiotic specific recombinase that together with ubiquitously expressed RAD51 catalyzes homologous pairing and DNA strand exchange [30]–[31]. These early steps in recombination are critical for establishing the physical connections between homologous chromosomes during meiosis. Hop1 has been implicated in modulating the formation and processing of double stranded breaks [19]. We examined formation of DMC1, RAD51, and RPA foci in zygotene stage *Hormad1^−/−^* spermatocytes. There is a dramatic decrease in the number of DMC1 foci as compared to the wild-type (Figure 5A and 5B). We counted a total of 98.9±28.2 DMC1 foci in the wild-type spermatocytes (n = 50) and 9.28±3.9 DMC1 foci in the *Hormad1^−/−^* spermatocytes (n = 45). The number of RAD51 foci was also decreased from 189.3±31.8 in the wild-type spermatocytes (n = 50), to 69.3±34.5 in the *Hormad1^−/−^* spermatocytes (n = 40) (Figure 5C and 5D).

Following double-strand breaks formation by SPO11, RPA is recruited together with RAD51 to the single stranded DNA regions [32]. We counted a total of 194.5±64.5 RPA foci in the wild-type spermatocytes (n = 30) as compared to 70.1±38.5 foci in the *Hormad1^−/−^* spermatocytes (n = 20) (Figure 5E and 5F). These results indicate that early meiotic recombinational events were disrupted and not surprisingly, MLH1, a protein that forms foci in later stages of recombination and required for the formation of most of the crossovers (chiasmata) observed in mice [33], was dramatically reduced in *Hormad1^−/−^* spermatocytes (data not shown).

**Figure 6:**
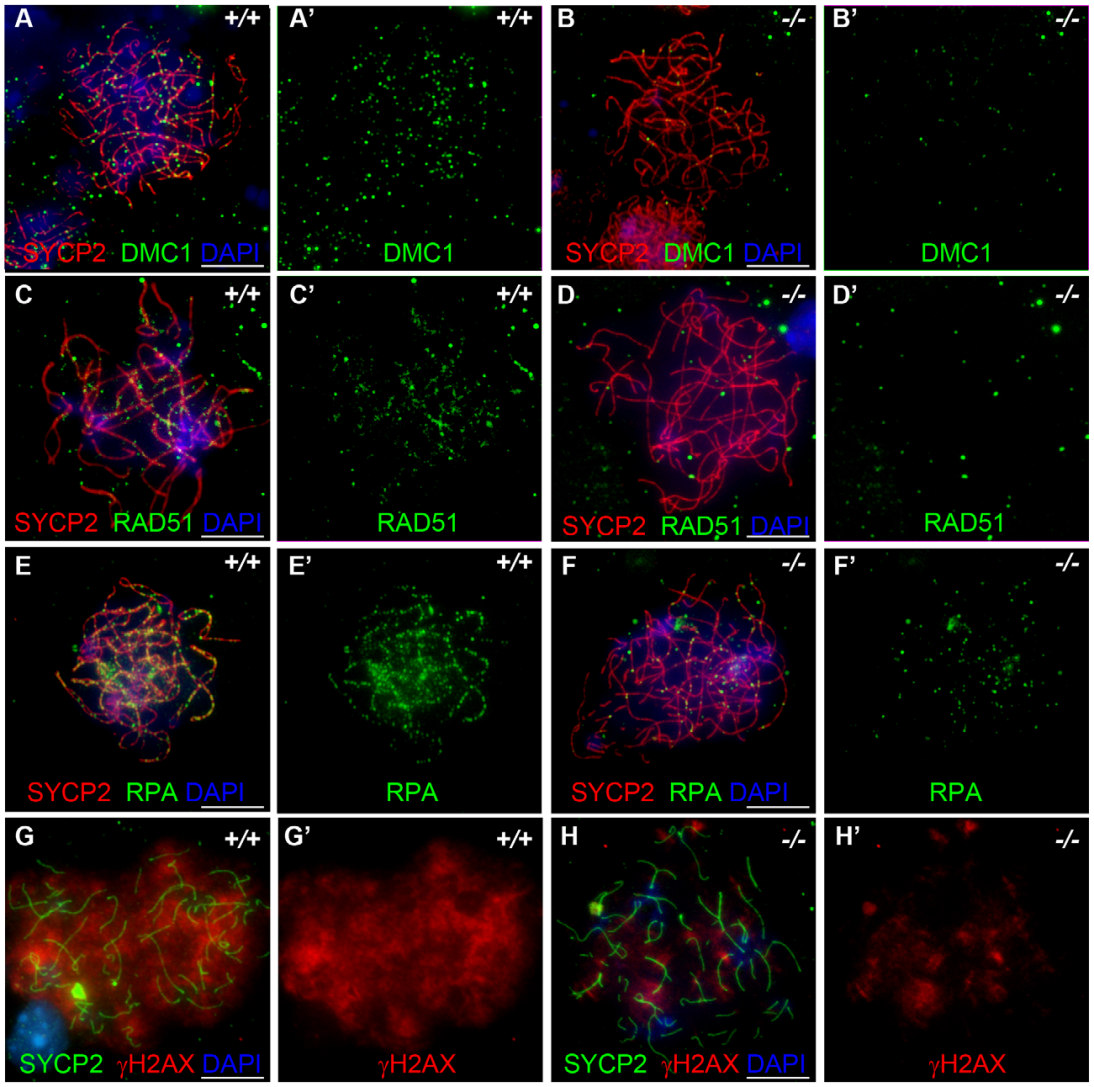
*Hormad ^−/−^* fetal oocytes are defective in early recombination. Chromosome spread assay in the wild-type and *Hormad1^−/−^* E15.5 fetal ovary. A-E represent zygotene stage, G and H represent leptotene stage. (A-B') Immunofluorescence assay with anti-SYCP2 (Red) and anti-DMC1 (Green) antibody. (C-D') Immunofluorescence assay with anti-SYCP2 (Red) and anti-RAD51 (Green) antibody. (E-F') Immunofluorescence assay with anti-SYCP2 (Red) and anti-RPA (Green) antibody. (G-H') Immunofluorescence assay with anti-SYCP2 (Green) and anti-γH2AX (Red) antibody. DMC1, RAD51, RPA and γH2AX signals were significantly decreased in *Hormad1^−/−^* fetal oocyte. DNA was stained with DAPI (Blue). Scale bars: 10 µm.

We also examined DMC1, RAD51 and RPA foci formation in female meiocytes at embryonic day 15.5 (E15.5). Embryonic ovaries contain zygotene to early pachytene oocytes at E15.5 [34]. We counted a total of 208.7±117.1 DMC1 foci in the wild-type E15.5 oocytes (n = 50), and 79.1±81.5 foci in the *Hormad1^−/−^* oocytes (n = 30) (Figure 6A and 6B), a total of 197.9±46.0 RAD51 foci in the wild-type oocytes (n = 50) versus 85.1±37.6 foci in the *Hormad1^−/−^* oocytes (n = 40) (Figure 6C and 6D) and a total of 317.16±135.3 RPA in the wild-type oocytes (n = 50) and 51.7±48.8 in the *Hormad1^−/−^* oocytes (n = 50) (Figure 6E and 6F). DMC1, RAD51 and RPA foci are therefore, similar to our observations in spermatocytes, significantly decreased in *Hormad1* deficient female meiocytes.

**Figure 7:**
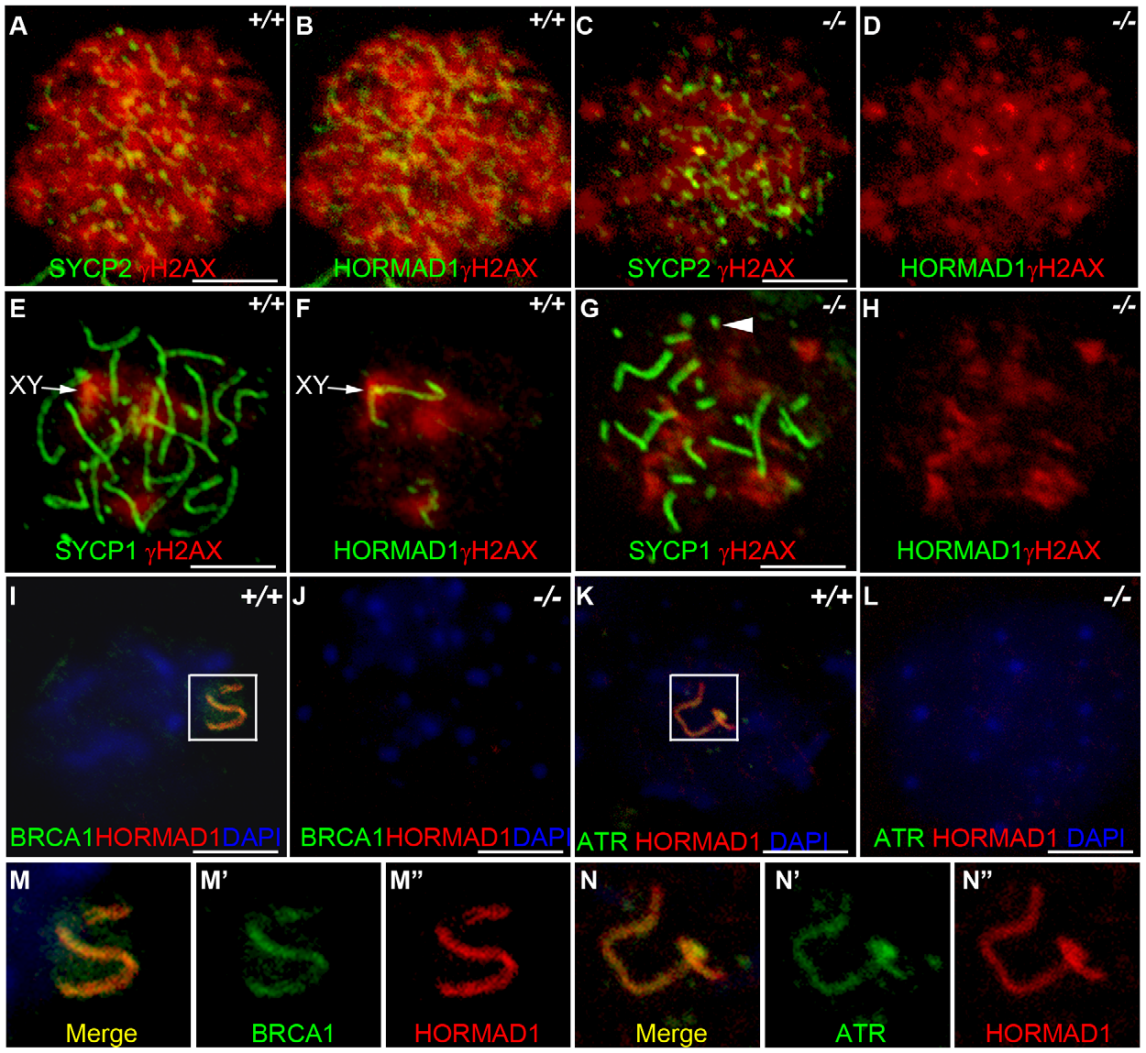
HORMAD1 disrupts γH2AX, BRCA1, and ATR localization to the XY chromosomes. Chromosome spread assay was performed on wild-type and *Hormad1^−/−^* leptotene (A-D), zygotene (E-H) and pachytene (I-L) spermatocytes to determine effects of HORMAD1 deficiency on γH2AX, BRCA1 and ATR localization to the sex chromosomes. (A-D) Immunofluorescence assay with anti-SYCP2 (Green) or anti-HORMAD1 (Green) and anti-γH2AX (Red). (E-H) Immunofluorescence assay with anti-SYCP1 (Green) or anti-HORMAD1 (Green) and anti-γH2AX (Red) antibody. (C and D) At the leptotene stage, γH2AX and SYCP2 localization to chromatin can be detected in *Hormad1^−/−^* spermatocytes, however, γH2AX is substantially reduced. (F and H) γH2AX staining localizes preferentially to the sex chromosome in the wild-type pachytene stage spermatocytes but no preferential localization to the sex chromosomes was observed in the *Hormad1^−/−^* spermatocytes, which is not surprising since sex body does not form in *Hormad1* mutants. (G) Arrow head indicates truncated axial fiber. (I, M-M”) BRCA1 and HORMAD1 co-localize to the sex chromosomes, but *Hormad1* deficiency disrupts BRCA1 localization (J). Similarly, ATR co-localizes with HORMAD1 to the XY chromosomes (K, N-N”), and *Hormad1* deficiency disrupts ATR localization (L). These results indicate that HORMAD1 is upstream of the currently known critical components of the meiotic sex chromosome inactivation complex, γH2AX, BRCA1 and ATR. DNA was stained with DAPI (Blue), and arrows indicate sex chromosomes. Scale bars: 10 µm.

These results indicate that homologous recombination is significantly affected in *Hormad1^−/−^* mammalian germ cells, as previously reported for *HOP1* [35]. We also observed effects of *Hormad1* deficiency on γH2AX staining (a phosphorylated form of histone H2AX), a well known surrogate marker for DSB formation [36]. In the leptotene stage, phosphorylation of H2AX is induced by SPO11 catalyzed DSBs in meiotic DNA, and γH2AX appears as large, cloud-like patterns thatdisappearat the pachytene stage [37]. At the leptotene stage, γH2AX staining in *Hormad1^−/−^* spermatocytes was significantly decreased (76% decrease in signal intensity) as compared to the wild-type (Figure 7A–7D). γH2AX staining was also significantly decreased in *Hormad1^−^* ^/*−*^ fetal oocytes (71% decrease in signal intensity) (Figure 6G and 6H). These results suggest that similar to Hop1 mutants, DSBs do not efficiently form in *Hormad1* mutants.

### HORMAD1 is a germ cell–specific component of the meiotic sex chromosome inactivation complex

HORMAD1 protein localization along autosomes was previously shown to be transient [17] (Figure 7B and 7F). HORMAD1 staining was highest along unsynapsed chromosome axis in the zygotene to pachytene stage and diminished significantly along autosomes in the pachytene [17]–[18] (Figure 7F and 7I). Interestingly, HORMAD1 localized strongly along desynapsed autosomes in diplotene meiocytes [17]. During the pachytene stage, HORMAD1 is faintly visible along the autosomes, but co-localizes strongly with γH2AX on the XY chromosomes, and specifically along the axial elements [17]–[18]. γH2AX is a phosphorylated form of histone H2AX, and a marker for DSBs [36], [38]. H2AX is phosphorylated throughout the chromatin in leptotene spermatocytes and by the pachytene stage [17]–[18], γH2AX staining is undetectable on autosomes and restricted to the sex body (Figure 7E and 7F) [39]. *H2ax* knockout shows the essential role for H2AX in sex body formation and meiotic sex chromosome inactivation [39]. Meiotic sex chromosome inactivation also involves ATR and BRCA1 dependent phosphorylation of H2AX [18], [40]. Interestingly, *Hormad1* deficiency, similar to *H2ax* deficiency, abolishes the formation of the sex body (Figure 7G and 7H, and data not shown). The lack of sex body formation is most likely due to the disruption of *Hormad1^−/−^* spermatocytes prior to the pachytene. We examined the γH2AX localization in the wild-type and *Hormad1^−/−^* spermatocytes. Chromatin in *Hormad1^−/−^* spermatocytes stained with anti-γH2AXantibodies, but no preferential localization to the sex chromosomes was observed (Figure 7G and 7H). BRCA1 and HORMAD1 have recently been shown to co-localize in the sex body [18]. We determined whether BRCA1 localization is dependent on HORMAD1. BRCA1 protein could not be localized in *Hormad1^−/−^* spermatocytes (Figure 7I–7J, 7M). This finding is unlike *H2ax* knockout, where BRCA1 is still detected on the sex chromosomes despite the lack of the sex body [40]. We also examined ATR localization in wild-type and *Hormad1^−/−^* testes. In wild-type testes, HORMAD1 and ATR co-localized in the sex body, but we could not detect ATR in *Hormad1^−/−^* spermatocytes (Figure 7K–7L, 7N). Above data suggest that HORMAD1 may be involved in the recruitment of BRCA1, ATR and γH2AX to the sex chromosome.

**Figure 8:**
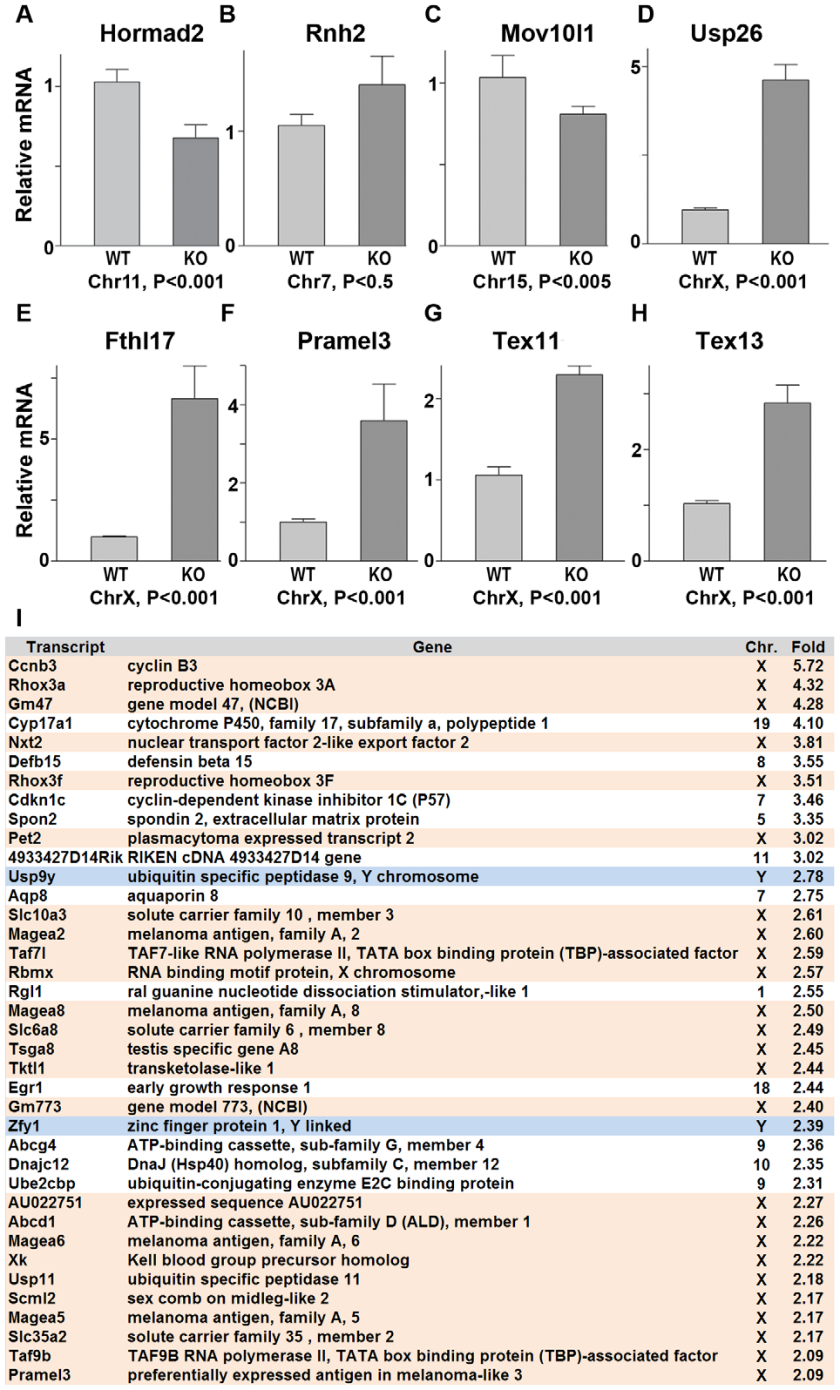
Sex chromosome expressed transcripts escape meiotic sex chromosome inactivation in *Hormad ^−/−^* spermatocytes. (A–H) Quantitative real time PCR analyses show that expression of testes specific genes derived from the autosome, *Hormad2*, *Rnh2*, and *Mov10l1*, did not significantly differ between the wild-type and the *Hormad1^−/−^* testes (A-C). However, X chromosome linked, testis specific genes, *Usp26*, *Fthl17*, *Pramel3*, *Tex11* and *Tex13* transcript were 2–6 fold increased (D-H). (I) Microarray analysis shows preponderance of X and Y derived transcripts among the top 38 up-regulated genes in *Hormad ^−/−^* testes. Data are normalized to *β-actin* expression and presented as the mean relative quantity (compared to wild-type) with error bars representing the standard error of mean. Student's *t* test was used to calculate the *P* values.

Since ATR, BRCA1 and γH2AX are involved in the transcriptional silencing of sex chromosomes, we examined whether *Hormad1* deficiency affects transcriptional repression. Previous studies have shown that *H2ax* and *Brca1* deficiencies individually, lead to the over-expression of genes exclusively expressed from the X or Y chromosome [40]. We examined whether X-linked germ cell–specific genes were over-expressed in *Hormad1^−/−^* testes compared to wild-type testes. We performed quantitative real-time PCR on select autosomal genes (*Hormad2*, *Rnh2* and *Mov10l1*) as well as X-chromosome derived genes (*Usp26*, *Fthl17*, *Pramel3*, *Tex11*, and *Tex13*) on wild-type and *Hormad1^−/−^* testes (Figure 8A–8H). *Rnh2* and *Mov10l1* are germ cell–specific transcripts, derived from autosomes, and were not differentially expressed between wild-type and *Hormad1^−/−^* testes (Figure 8B and 8C). In contrast, all of the germ cell–specific transcripts transcribed from the X chromosome were significantly elevated in *Hormad1^−/−^* testes over the wild-type. These include *Usp26* (4.5 fold increase), *Fthl17* (6.5 fold increase), *Pramel3* (3.5 fold increase), *Tex11* (2.2 fold increase), and *Tex13* (2.8 fold increase) (Figure 8D–8H). Moreover, RNA expression microarray analyses comparing two week old *Hormad1* deficient testes with corresponding wild-type, indicate that almost 20% of the up-regulated genes derive from the X chromosome (Figure 8I). Our results are remarkably similar to transcriptional de-repression observed in *H2ax* and *Brca1* mutants [39]–[40], and indicate that HORMAD1 is a germ cell and meiosis specific factor critical in meiotic sex chromosome inactivation and transcriptional silencing.

### *Hormad1* deficiency abrogates ATM autophosphorylation

**Figure 9:**
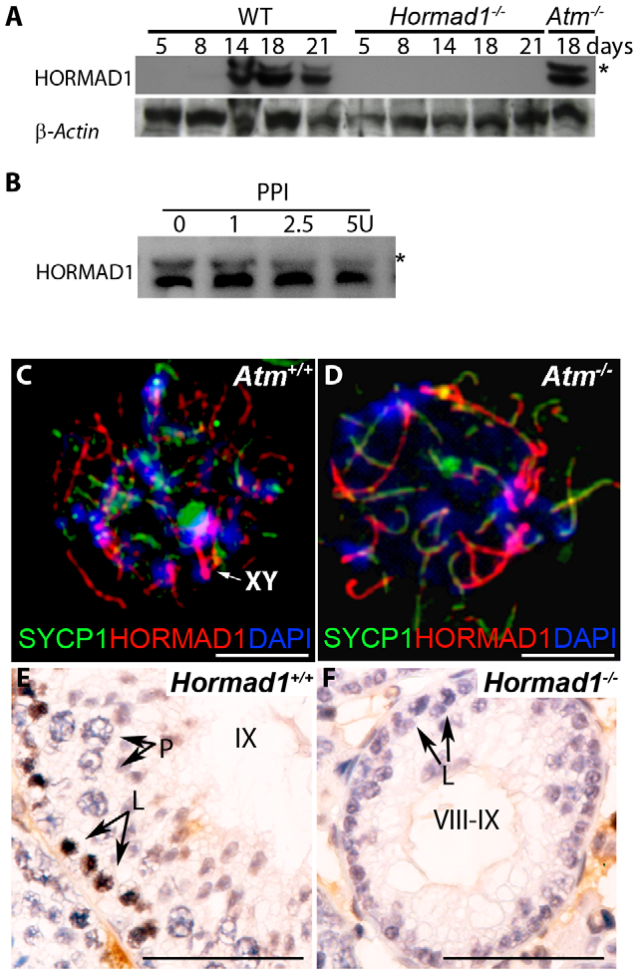
HORMAD1 phosphorylation is unaffected by *Atm* deficiency while *Hormad1* deficiency disrupts ATM autophosphorylation. (A) Western blots analyses with anti-HORMAD1 specific antibodies show that HORMAD1 protein and its phosphorylated form (*) appear circa post-natal day 14. *Atm1* deficient testes (*Atm^−^* ^/*−*^) express both forms of HORMAD1 and HORMAD1 is therefore unlikely to be ATM1 substrate. (B) The presumed phosphorylated form of HORMAD1 (higher molecular weight band indicated by asterisk) decreases in intensity after protein phosphatase I (PPI) treatment. (C and D) Chromosome spread assay in wild-type (*Atm^+/+^*), and *Atm* deficient spermatocytes (*Atm^−/−^*). HORMAD1 preferentially localizes to unsynapsed regions of chromosome axes (C), and HORMAD1 antibody stains unsynapsed chromosome axes in *Atm^−/−^* spermatocytes (D) more intensely than in the wild-type spermatocytes. Scale bars: 10 µm. (E and F) Immunohistochemistry analysis in 6 week old wild-type and *Hormad1^−/−^* testes with anti-phospho ATM-S1981 antibody. Phospho-ATM S1981 was detected in wild-type zygotene, but not detected in *Hormad1^−/−^* zygotene spermatocytes. Scale bars: 50 µm.

HORMAD1 is a likely mammalian counterpart to the yeast HORMA domain meiotic protein, Hop1. Hop1 is phosphorylated by Mec1/Tel1, the budding yeast homologue to the mammalian ATR and ATM kinases, and this phosphorylation is thought to play an important role in inter-homologous recombination [21]. We therefore examined HORMAD1 expression in *Atm* deficient mice as well as ATM expression in *Hormad1^−/−^* animals. ATM is a serine/threonine-specific protein kinase that has been associated with cell cycle regulation, apoptosis, and response to DNA damage repair. ATM kinase activation is associated with increased auto-phosphorylation of ATM at multiple sites including serine 1981 [41]. We examined HORMAD1 protein expression in testes between postnatal days 5–21 (Figure 9A). Eight to ten day old testes contain spermatogonial cells as well as preleptotene/leptotene spermatocytes [34]. At postnatal day 14–18, testes contain pachytene spermatocytes and visible sex bodies [34]. HORMAD1 is known to be phosphorylated [17] (Figure 9B). Western blot analysis on testes extracts detected phospho-HORMAD1 beginning at postnatal day 14 (Figure 9A). Phospho-HORMAD1 protein was decreased in postnatal day 18 and 21 wild-type testes (Figure 9A). Phospho-HORMAD1 appearance correlates temporally with sex body formation. HORMAD1 phosphorylation was not affected by *Atm* deficiency (Figure 9A). These results indicate that ATM is not responsible for HORMAD1 phosphorylation. We also examined HORMAD1 localization in *Atm^−/−^* spermatocytes. We observed HORMAD1 localization to chromosomal axes in *Atm* deficient spermatocytes, as previously described by others [17] (Figure 9C and 9D). Since synaptonemal complexes do not form in *Atm* mutants, HORMAD1 association with unsynapsed chromosomes does not require ATM. The anti-ATM phospho-S1981 antibody did not detect phosphorylated ATM in *Hormad1^−/−^* testes (Figure 9E and 9F). These results suggest that HORMAD1 is upstream of ATM auto-phosphorylation, and therefore likely upstream of ATM kinase activation.

### *Hormad1* deficiency does not affect gross ovarian development

**Figure 10:**
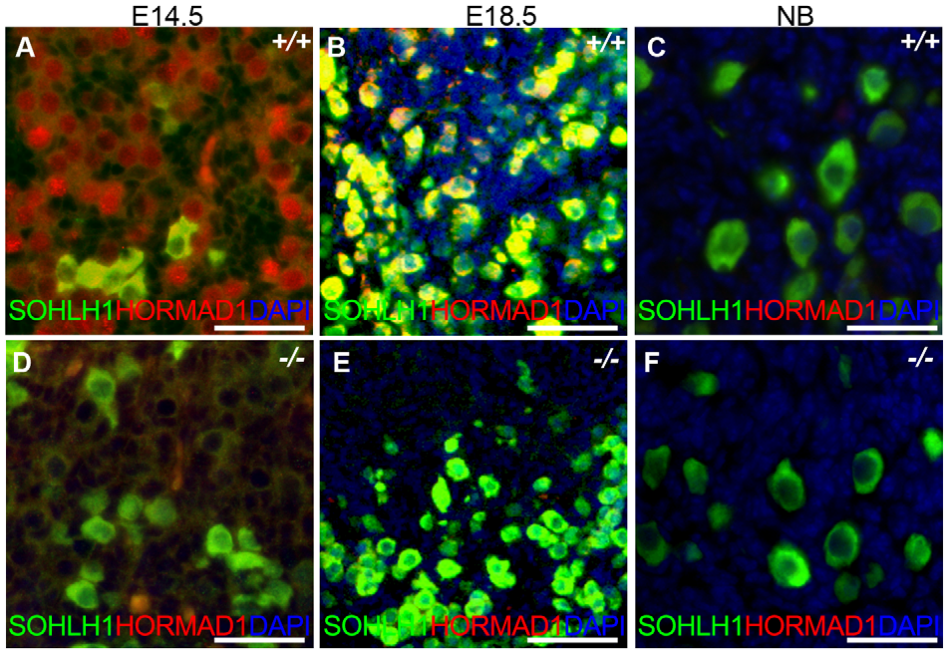
HORMAD1 is expressed in embryonic but not post-natal oocytes. (A–C) HORMAD1 protein was detected by immunofluorescence in the embryonic ovary at E14.5 (A), E18.5 (B), but not in the newborn ovary (C). (D–F) Oocytes in *Hormad1^−/−^* ovary (*−*/*−*) were stained with antibodies against the germ cell–specific transcriptional regulator SOHLH1 at E14.5 (D), E18.5 (E), and newborn ovary (F). DNA was stained with DAPI (Blue). Scale bars: 20 µm.

We have previously shown that *Hormad1* RNA expression in the ovary was confined to the germ cell [16]. Meiosis I in the female gonad commences circa E13.5 and most oocytes arrest at the dictyate stage by the time of birth. Antibodies against HORMAD1 recognized HORMAD1 protein at E14.5 (leptotene) and E18.5 (arrest in diplotene) oocytes (Figure 10A and 10B), but little HORMAD1 protein was detected in the newborn ovary oocytes (Figure 10C), at the time when oocytes are arrested in diplotene. Deficiency in genes critical in meiosis can disrupt early ovarian development, as is the case for *Dmc1*, *Msh5*, *Spo11* and *Atm* [42]–[44].

**Figure 11:**
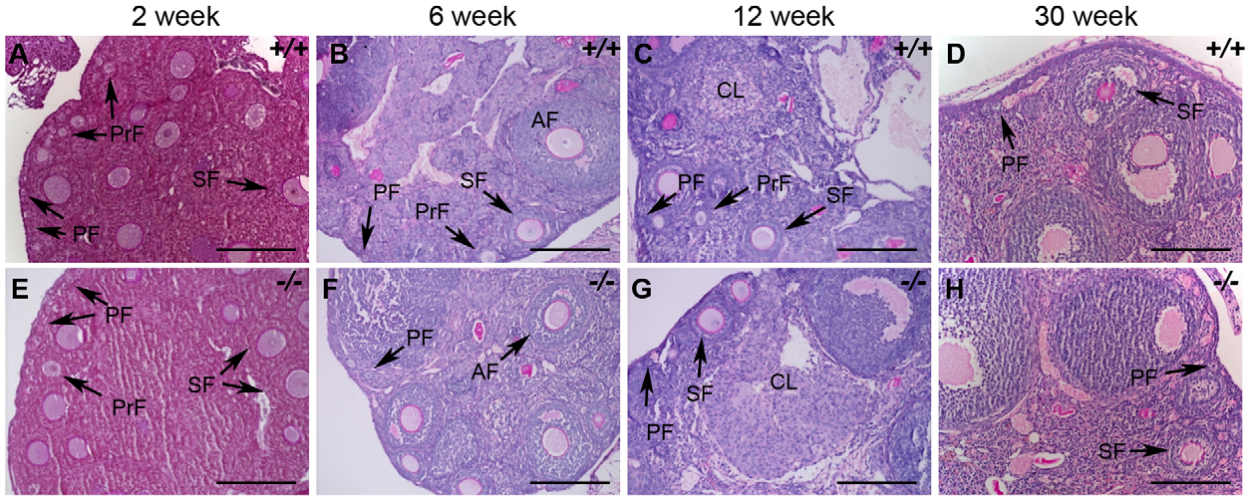
Histological analysis of wild-type and *Hormad1* ^–/–^ ovaries. (A–H) PAS staining of wild-type (+/+) and *Hormad1^−/−^* (*−*/*−*) ovaries does not show gross histological differences. Wild-type and *Hormad1^−/−^* ovaries are shown at different post-natal ages that show the full range of ovarian follicles: primordial follicles (PF), primary follicles (PrF), secondary follicles (SF), antral follicles (AF) and corpus luteum (CL). Scale bars: 50 µm.

We therefore examined ovarian development in *Hormad1^−/−^* females. Antibodies against germ cell–specific transcriptional regulator SOHLH1 [45] stained wild-type and knockout oocytes throughout embryonic gonadal development with no significant differences noted (Figure 10A–10F). Moreover, the numbers of primordial, primary and secondary follicle counts did not significantly differ between the mutant and wild-type ovaries at post-natal day 8 (Figure S4). These results indicate that *Hormad1* deficiency does not affect embryonic ovarian development, germ cell cyst breakdown, and primordial follicle formation. We also examined the histology of wild-type and *Hormad1^−/−^* ovaries between 2 and 30 weeks of life. Mice reach sexual maturity around 6 weeks of life, and mouse ovaries at this time consist of all of the follicular types including corpora lutea, an indication that the ovaries are ovulating. Postnatal *Hormad1^−/−^* ovaries were grossly indistinguishable from wild-type mice between 2 and 30 weeks of life, with abundant corpora lutea in the *Hormad1^−/−^* ovaries indicating that the normal process of oocyte maturation was not disrupted, and ovulation has occurred (Figure 11A–11H). We induced superovulation in knockout and wild-type mice with exogenous gonadotropins to determine whether subtler ovarian defects contributed to infertility in *Hormad1^−/−^* mice. *Hormad1^−/−^* females super-ovulated 28±11 eggs (n = 22), while wild-type animals superovulated 29±14 eggs (n = 13). We therefore did not observe significant difference between the number of eggs superovulated from wild-type versus knockout mice. These results indicate that ovarian development is grossly normal in *Hormad1^−/−^* mice, and that ovarian defects are unlikely to account for observed infertility.

### *Hormad1* deficient eggs fertilize and embryonic development arrests at blastocyst stage due to aneuploidy

**Figure 12:**
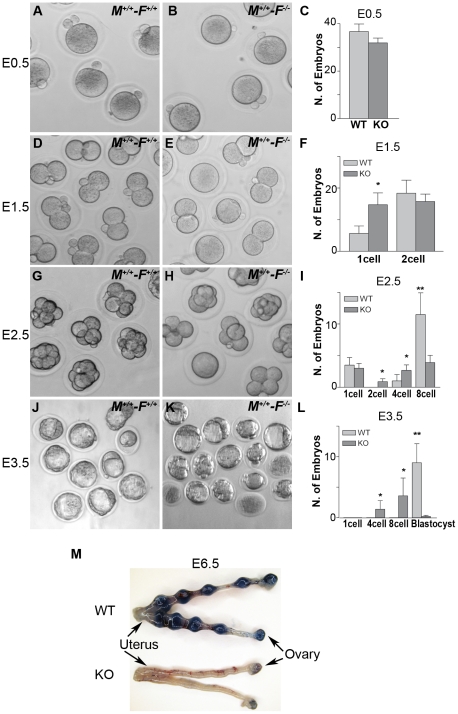
*Hormad1^–/–^* oocyte derived embryos arrest at blastocyst stage. Embryos derived from wild-type matings (M^+/+^-F^+/+^) and matings involving *Hormad1^−/−^* oocytes fertilized by wild-type males (M^+/+^-F*^−^* ^/*−*^), were collected at E0.5, E1.5, E2.5 and E3.5 for the analysis of the early embryonic development. (A-C) No significant differences were noted between wild-type and *Hormad1^−/−^* at E 0.5, with normal appearing one cell embryos and polar bodies visible in both. (D-F) At E1.5, there is an excess of 1 cell embryos in *Hormad1^−/−^* fertilized oocytes, indicating a lag in embryo development at this stage. (G-I) At E2.5 there is a significantly less number of eight cell embryos formed in *Hormad1^−/−^* fertilized oocytes as opposed to the wild-type. A large proportion of 8-cell embryos appear disorganized and apoptotic. (J-L) Blastocysts form at E3.5 in wild-type but very few normal blastocysts are visible in *Hormad1^−/−^* embryos. The count only includes morphologically normal blastocysts (L). Error bars represent standard error of the mean. Student's *t* test was used to calculate *P* values (*:*P*<0.5, **:*P*<0.001). (M) Embryos derived from *Hormad1^−/−^* fertilized oocytes can not implant, as visualized by Sky Blue dye injected into the tail vein of pregnant females at E6.5 gestational age.

We studied early embryonic development of fertilized *Hormad1^−/−^* oocytes because ovarian defects were unlikely to explain observed infertility. We recovered embryos from wild-type male matings with *Hormad1^−/−^* females at E0.5, E1.5, E2.5, and E3.5. Comparable numbers of morphologically indistinct 1-cell zygotes were recovered from oviducts of control and mutant female mice at E0.5 (Figure 12A–12C), and little difference was noted at the 2-cell stage, except for an increased number of 1-cell embryos in the knockouts, indicating a lag in the progression from the 1-cell to 2- cell stage (Figure 12D–12F). By E2.5, the number of normal appearing 8-cell stage embryos was significantly less in *Hormad1^−/−^* fertilized eggs as opposed to the wild-type (Figure 12G–12I), and no morphologically normal blastocysts were observed in the *Hormad1^−/−^* fertilized eggs at E3.5. It is interesting to note that at E3.5, a significant number of 4 and 8 cell stage embryos were observed in the knockout while only blastocysts were observed in the wild-type E3.5 embryos (Figure 12J–12L). We also tested, using Chicago Sky Blue 6B dye (Sigma, MO, USA) injection into the tail vein [46], whether blastocysts derived from *Hormad1^−/−^* fertilized eggs could implant. We did not detect implantation of *Hormad1^−/−^* fertilized eggs (Figure 12M). These results indicate that a defect in early embryogenesis led to premature loss of embryos.

### *Hormad1* deficiency causes aneuploidy and early embryonic demise

**Figure 13:**
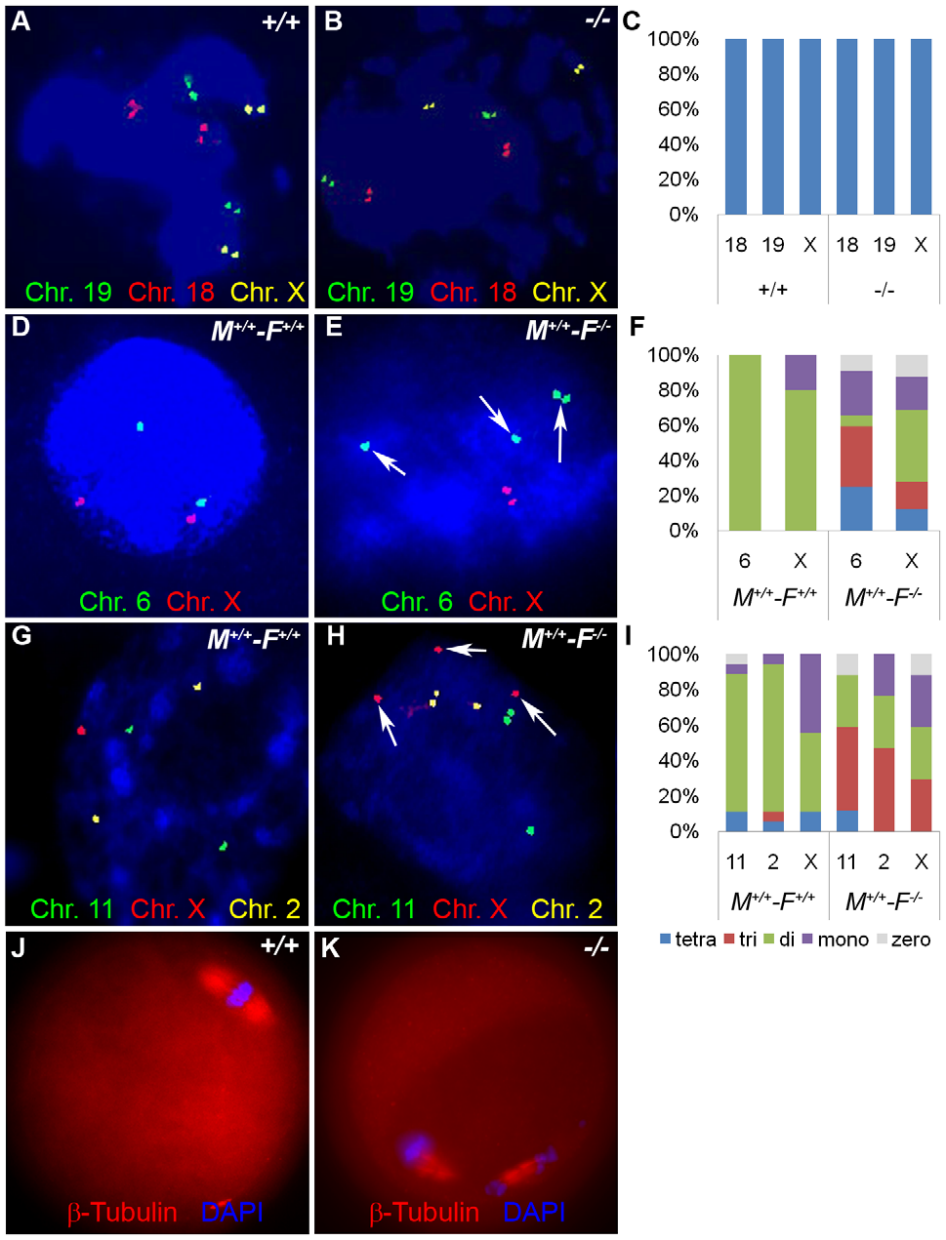
*Hormad1^–/–^* fertilized oocytes show widespread hypo- and hyperdiploidy. (A-B) In GV stage oocytes, parental chromosomes have replicated to produce identical sister chromatids, which are visualized as closely located double signals for examined chromosomes 18, 19 and X. (C) Histogram of individual chromosomes, does not show significant difference between the wild type and mutants. Fifty eight knockout GV oocytes and 112 wild type GV oocytes were scored. Abbreviations stand for the following: tetra-Tetrasomy (blue), tri-Trisomy (red), di-Disomy (green), mono-Monosomy (purple), zero-Zerosomy (grey). (D-F) E1.5 day (2-cell stage embryo) FISH assay using chromosomes 6 (green) and X-specific DNA probes (red). The wild type embryos were diploid for 6 and X (D). Trisomy for chromosome 6 (arrows) is noted in the knockout (E). In addition, chromosomes 6 and X demonstrate double hybridization signals, the same FISH pattern as observed during the GV stage, and indicative of sister chromatids. (F) Histograms for individual chromosomes show high prevalence of chromosomal aneuploidies in embryos derived from mutant oocytes. We scored 63 knockout and 46 wild type cells. (G-I) E2.5 day (8-cell stage embryo) FISH assay with chromosomes X (red), 11 (red) and 2 (yellow) specific probes. (G) A hybridization pattern of a normal diploid number of chromosomes in the wild type embryo. (H) Trisomy for chromosome X (arrow), as well as double signals likely produced by sister chromatids on chromosome 2 and 11 were observed in embryos derived from knockout eggs. (I) Histogram shows substantial number of trisomies and monosomies in the knockout as compared to the wild type. We scored 84 wild type and 96 knockout cells. (J and K) Immunofluorescence with β-tublin antibody (red) in wild-type and *Hormad ^−/−^* MII stage oocytes. *Hormad ^−/−^* oocytes are grossly disrupted with mis-orientation of the chromatid attachment to the spindle. DNA was stained with DAPI (Blue).

We hypothesized that aneuploidy is the major cause of embryo wastage in *Hormad1^−/−^* females. Unlike meiosis in testes, errors during oocyte meiosis have milder effect on oocyte loss and apoptosis, as has been observed in *Sycp3* and *Smc1β* knockouts [27], [29], [47]. The presence of growing oocytes in the *Hormad1^−/−^* ovaries, as opposed to early germ cell loss observed in testes during pachytene stage of meiosis, indicates greater tolerance of *Hormad1* deficiency in the oocytes as compared to spermatocytes. The whole range of ovarian follicle types are present in the *Hormad1^−/−^* ovaries, including primordial follicles, primary, secondary and antral follicles. In contrast to other critical genes during meiosis, such as *Dmc1*, *Msh5*, *Spo11* and *Atm* that cause early ovarian failure due to rapid loss of oocytes following disruption of meiosis I [42]–[44], *Hormad1* deficiency does not activate apoptotic pathways and does not lead to gross premature loss of oocytes. We used chromosome specific mouse BAC (bacterial artificial chromosome) clones to perform fluorescent *in situ* hybridization (FISH) on germinal vesicle (GV) oocytes, 2 cell and 8 cell stage embryos to determine whether aneuploidy is significantly higher in *Hormad1^−^* ^/*−*^ embryos. Wild-type GV oocytes are arrested in meiosis I and contain bivalent chromosomes that consist of four chromatids. Examination of 58 GV oocytes from three independent knockout animals and 112 GV oocytes from three wild-type animals with BAC probes specific for chromosomes 19, 18 and X, revealed no significant differences, with presence of four chromatids for each of the chromosome examined as expected (Figure 13A–13C). Following fertilization and completion of meiosis II, each cell in the developing embryo should contain two chromosomes except for the sex chromosomes. We examined by FISH, mouse chromosomes 6 and X in the 2-cell wild-type and *Hormad1^−/−^* embryos. Sixty three cells examined for chromosome 6 in the *Hormad1^−/−^* 2-cell embryos revealed that 22% of the cells had 4 signals corresponding to chromosome 6, 35% had 3 signals corresponding to chromosome 6, 24% had 1 signal, and 5% had no signal. Wild-type 2-cell embryos were also examined by chromosome specific FISH, and 44 cells examined out of 46 showed only 2 signals, as expected. In 2-cell *Hormad1^−/−^* embryos, out of sixty three cells examined for chromosome X, 44% showed four, three or no signal. Wild-type 2-cell stage embryos, as expected, showed only 2 or 1 signal, consistent with either XX or XY sex of the cell. These results indicate widespread hypo and hyperdiploidy in 2-cell embryos (Figure 13D–13F).

We also examined cells from the 8-cell stage embryos. A total of 84 cells from the *Hormad1^−/−^* embryos were examined with FISH probes specific for chromosomes 11, X and 2. *Hormad1^−/−^* cells from the 8-cell stage, hybridized with BAC specific to chromosome 11, showed 4 signals in 10% of the cells, and 3 signals in 55% of the cells. Therefore, a total of 65% of the cells were either monosomic or trisomic for chromosome 11. In the wild-type 8 cell stage embryo, a total of 96 cells were scored for chromosome 11, and only 2 out of 96 (2%) cells showed a single signal, while 94 out of 96 cells (98%) showed two signals as expected. Similar results were obtained for chromosome 2 (Figure 13G–13I). Our results indicate widespread aneuploidy involving different chromosomes in both 2-cell and 8-cell embryos. Such aneuploidy causes early embryo demise and failure of implantation.

We also visualized second metaphase (M2) spindle using anti-β-tubulin antibodies in *Hormad1^−/−^* oocytes. During M2, sister chromatids migrate to the opposite pole to form functional, haploid gametes. Sister chromatids are bi-oriented on the spindle and congregate in the center of the spindle prior to separation (Figure 13J). Each sister kinetochore in a pair is attached to the opposite spindle, and such arrangement generates sufficient tension to cause proper segregation. M2 in *Hormad1^−/−^* oocytes is grossly disrupted with mis-orientation of chromatid attachment to the spindle observed in all M2 oocytes examined (Figure 13K) (n = 50). These results indicate importance of *Hormad1* in proper spindle formation and segregation.

## Discussion

We discovered *Hormad1* (*Nohma*), using *in silico* screen to identify germ cell–specific transcripts critical for gonadal development [16]. We showed that *Hormad1* expression was confined to germ cells, and that *Hormad1* transcripts were mostly concentrated in the pachytene spermatocytes and early oocytes. Here we show that *Hormad1* is essential for both male and female fertility in the mouse. HORMA domain-containing proteins interact with chromatin, particularly chromatin associated with DNA adducts, and are critical mitotic spindle checkpoint proteins [5]. HORMA domain-containing proteins are critical regulatory proteins for mitosis as exemplified by MAD2 protein, and meiosis as exemplified by Hop1p [8], and Red1p [9] in yeast, Him-3 [10] in nematodes, and Asy1 [11] in plants. The 205-aa HORMA domain in the mouse HORMAD1, shares highest similarity to the *Saccharomyces cerevisiae* Hop1 protein (28% amino acid similarity). Yeast *Hop1* mutants have defects in chromosome condensation, synapsis and recombination [4], [8], and Hop1p binds DSBs during meiotic prophase and appears to play an important role in interhomologue recombination. We previously hypothesized, based on homology to Hop1 in yeast, that *Hormad1* plays an important role in mammalian meiosis. Our results indicate that HORMAD1 is the mammalian homologue to the Hop1 protein in yeast.

Our current studies, and those of others, have shown that HORMAD1 protein is confined to germ cells and co-localizes with synaptonemal complex proteins SYCP2 and SYCP3 on the chromatin as part of the axial core [27], [48]. Synaptonemal complex is a complex structure composed of multiple germ cell–specific and ubiquitously expressed proteins that connect paired homologous chromosomes. Similar to railroad tracks, the synaptonemal complex axial lateral elements (SYCP2, SYCP3) are connected to each other by proteins known as transverse filaments (SYCP1, TEX12) [1]. The axial/lateral elements play critical roles in chromosome condensation, pairing, and repress recombination pathways that involve sister chromatids [1]. Synaptonemal complex formation is associated with HORMAD1 depletion from the axes [17]–[18]. Our data show that HORMAD1 phosphorylation peaks at the time when HORMAD1 localization shifts to the sex body. ATM and ATR do not appear to be involved in HORMAD1 phosphorylation and binding to chromosomal axes. Previous study showed that Trip13, a homologue of yeast Pch2 kinase, may be involved in HORMAD1 depletion from synapsed chromosome axes [17]. Kinases, such as Mec1/Tel1 in yeast are important to effect inter-homologous recombination [21] by phosphorylating Hop1, but not much is known regarding HORMAD1. Future studies are necessary to understand HORMAD1 phosphorylation sites and responsible kinases.

Although HORMAD1 is not essential for the binding of well-characterized germ cell–specific synaptonemal complex proteins such as SYCP1, SYCP2 and SYCP3, HORMAD1 deficient spermatocytes are defective in synapsis and do not form recognizable synaptonemal complexes. *Hormad1* deficiency causes a male meiotic arrest, that is similar to male meiotic arrests observed in other components of the synaptonemal complex and recombination including: *Atm*, *Spo11*, *Sycp1*, *Sycp2*, *Sycp3* and *Dmc1* [27], [30]–[31], [48]–[50]. These findings are not surprising as HORMAD1 co-immunoprecipitates with known axial proteins, SYCP2, SYCP3, REC8, and SMC1β [18]. Whether these interactions are biologically significant, and whether HORMAD1 interacts with other members of the synaptonemal complex, including newly discovered HORMAD 2 protein, is unclear. Interestingly, Hop1 interacts with another phosphoprotein, Red1 [35], [51], and whether HORMAD2 is the mammalian counterpart of Red1 remains to be seen.

Like its counterpart in yeast, *Hop1*, *Hormad1* deficiency disrupts early and later stages of recombination as shown by the drastic diminution in the formation of DMC1, RAD51, RPA and MLH1 foci. These findings differ from the *Sycp3* mutant, which affects later stages of recombination [29]. DMC1 is a meiotic specific recombinase that together with ubiquitously expressed RAD51, catalyzes homologous pairing and DNA strand exchange, and marks earlier stages of recombination than MLH1 foci [30]. HORMAD1 expression is not significantly affected in *Dmc1* mutant spermatocytes [17]. These results indicate that unrepaired DSBs and synaptonemal complexes between non-homologous chromosomes seen in *Dmc1* mutants, do not affect HORMAD1 localization. In *Hop1* mutants, DMC1 foci are only faintly detected when compared to controls [19]. These findings have led to a suggestion that Hop1 binds at or near the sites of DNA double strand break and modulates the action and perhaps recruitment of recombinases such as DMC1 and RAD51 [19]. Moreover, Hop1 appears to be part of the meiosis specific surveillance system that monitors meiotic double stranded break repair [21]. Our observations support the possibility that HORMAD1 affects early recombination and may therefore perform similar functions in the mammalian meiosis.

During mammalian meiosis, X and Y chromosomes are inactivated and transcriptionally silenced (meiotic sex chromosome inactivation, MSCI) and chromatin condenses to form a sex (XY) body. The epigenetics of MSCI involves γH2AX, BRCA1 and ATR at a minimum. The most intriguing finding regarding HORMAD1 is its co-localization with BRCA1, ATR and γH2AX in the sex chromosomes during pachytene [18]. *Hormad1* deficiency, similar to *H2ax* deficiency, abrogates formation of the sex body, as well as γH2AX localization to the sex chromosomes. *Hormad1* deficiency also abrogates BRCA1 and ATR localization to the sex chromosomes. Interestingly, *H2ax* deficiency does not affect BRCA1 localization to the sex chromosome, despite anomalous sex body, while BRCA1 deficiency does affect both ATR and γH2AX localization to the sex body. These findings indicate that HORMAD1 is involved in the initial recruitment of BRCA1, ATR and γH2AX to the sex body. The mechanism whereby HORMAD1 affects ATR, γH2AX and BRCA1 localization is unclear, but our results are consistent with the conclusion that HORMAD1 is upstream of ATR, γH2AX and BRCA1, and establishes HORMAD1 as an essential component of the MSCI complex. Moreover, we have shown here that *Hormad1* deficient testes preferentially over-express X-linked genes as observed in mice deficient for other components of the MSCI complex such as γH2AX and BRCA1 [17]. Almost 20% of genes preferentially up-regulated from the *Hormad1^−/−^* testes are derived from the X chromosome. *Hormad1* is therefore the first germ cell–specific component known to play a role in MSCI.

Unlike males, meiosis I in females arrests during the embryonic development at the diplotene stage and is completed upon ovulation. The lack of dramatic germ cell apoptosis during ovarian follicle development has also been observed for *Sycp2 and Sycp3* deficient mice [48], [52], and reiterates the laxity of oocytes to early meiotic errors, as opposed to spermatogenesis, where meiotic check point causes the dramatic phenotype observed in male gonads. *Hormad1* deficiency does not affect folliculogenesis nor ovulation. These findings are unusual, given that *Hormad1* affects recombination, and recombination associated proteins DMC1 and MLH1 are important in early oogenesis. Unrepaired recombination intermediates or defects in homologous chromosome pairing and synapsis are likely causes of early oocyte loss [53]. *Spo11* deficient animals can ameliorate observed oocyte losses in recombination defective mutants, presumably due to the failure of *Spo11^−/−^* deficient mice to form DSBs that initiate meiotic recombination. *Spo11* deficiency does not affect HORMAD1 association with the chromosome axes [17], a finding similarly observed for Hop1 localization in *Spo11^−/−^* yeast [9]. Moreover, *Hop1* mutants are required for full levels of DSBs formation [37], [54]. It is therefore possible that early loss of oocytes in *Hormad1* deficient animals does not occur in part because unrepaired DSBs do not form. Reduced staining of γH2AX in *Hormad1* mutant leptotene stage oocytes argues that unrepaired DSB formation is indeed reduced. However, unlike *Spo11^−/−^* ovaries that have abnormal folliculogenesis, *Hormad1^−/−^* ovaries are not defective in folliculogenesis and other mechanisms must protect *Hormad1* mutant oocytes from early loss.

Infertility in *Hormad1^−/−^* females is due to early embryo demise. This is in stark contrast to males, where spermatocytes are eliminated due to the pachytene defect. The greater ability of oocytes to tolerate meiosis I errors is well known [55], and exemplified by *Sycp3* and *Smc1β* knockouts [52], [56], which have defects in synapsis and recombination. Mechanisms that underlie oocyte's insensitivity to such errors are not well understood [57]. Embryos derived from *Hormad1^−/−^* oocytes fertilized with wild-type sperm, cannot implant. Examination of post-fertilization events in fertilized *Hormad1^−/−^* oocytes, showed significant abnormalities in early embryo development, including growth lag, and severe disruption in development prior to the blastocyst stage. Very few blastocysts were produced in fertilized *Hormad1^−/−^* oocytes, and most were abnormal in morphology. We utilized FISH hybridization against specific chromosomes to show widespread hypo and hyperdiploidy during early embryonic development. These results are reminiscent of *Sycp3* and *Smc1β* knockout embryos. SYCP3 and SMC1β are involved in the synaptonemal complex formation, are germ cell–specific, and implicated in human aneuploidy. *Sycp3* deficient females are subfertile, but one third of the embryos die *in utero* due to aneuploidy [29]. *Smc1β* encodes a meiosis specific component of the cohesin complex, important in sister chromatid cohesion and recombination [58]. *Smc1β* deficient females are sterile, like *Hormad1^−/−^* females, and meiosis continues until metaphase II [59].

However, unlike *Smc1β^−^* ^/*−*^, *Hormad1^−/−^* ovaries do not lose oocytes by 6 months of age, and postnatal oocyte counts do not differ significantly from the wild type. *Sycp3^−/−^* ovaries show significant decline in the primordial follicle pool at postnatal day 8, indicating accelerated apoptosis of oocytes as germ cell clusters break down to form primordial follicles [52]. DSBs, as indicated by the assembly of the histone variant γH2AX, form in *Smc1β^−^* ^/*−*^ and *Sycp3^−/−^* meiocytes and may lead to higher rate of oocyte loss in these mutants [47], [52]. *Hormad1* deficient ovaries do not display loss of primordial follicles. *Hormad1^−/−^* ovaries may not be losing oocytes at an accelerated rate due to our observations that DSBs, as assessed by γH2AX staining, are substantially reduced in *Hormad1* mutants. Similar to *Hop1* mutants in yeast, lack of HORMAD1 may derepress *Dmc1*-independent inter-sister repair pathway, resulting in efficient DNA break repairs [21]. The reduced DSBs in *Hormad1^−/−^* meiocytes, and de-repression of *Dmc1*-independent inter-sister repair pathways, may not affect regular apoptotic pathways that eliminate substantial number of oocytes during germ cell clusters breakdown to form primordial follicles at the time of birth, resulting in no decrease in *Hormad1^−/−^* primordial oocyte numbers.

## Materials and Methods

### Animal breeding

All murine experiments were carried out on the 129S7/SvEvBrd x C57BL/6 hybrid background. Litters were weaned at 3 weeks and breeding pairs set up at 6 weeks of age. One mating pair was placed per cage and inspected daily for presence of litters. All experimental and surgical procedures complied with the Guide for the Care and Use of Laboratory Animals and were approved by the Institutional Agricultural Animal Care and Use of Committee of Baylor College of Medicine and the Institutional Animal Care and Use of Committee of University of Pittsburgh.

### Targeting construct, generation of transgenic mice, and genotyping

Targeting construct, electroporation and ES cell selection was done as previously described [60]–[61]. Exons 4 and 5 of the mouse *Hormad1* gene were replaced with a neomycin resistance gene flanked by 5.3 kb (5′) and 6.2 kb (3′) homologous sequences (Figure S1A). Targeted ES cells were verified by Southern blotting, and microinjected into the C57BL/6 blastocysts to produce chimeric mice that carried the mutation into the germ line. These mice were mated with C57BL/6 wild-type mice to generate *Hormad1* heterozygous animals that were subsequently crossed to produce F_2_ offspring for functional analysis. PCR genotyping was performed using the following primers: WT-forward (5′- TCAAGACCAACCTGGGCTAC -3′) and WT-reverse (5′- CCATGTGGGTTGTAGGGAGT -3′) to amplify a 196-nucleotide wild-type band, and KO- forward (5′- TCAAGACCAACCTGGGCTAC -3′) and KO- reverse (5′- GGGGAACTTCCTGACTAGGG -3′) to amplify a 505-nucleotide mutant band (Figure S1A).

### Histology and immunohistochemistry analysis

Testes were fixed in Bouin's solution (Sigma-Aldrich, MO, USA) and ovaries were fixed in 10% buffered formalin or 4% paraformaldehyde. Fixed tissues were embedded in paraffin, serially sectioned (5 µm) and stained with hematoxylin and eosin or with Periodic Acid Schiff (PAS). At least five pairs of testes and ovaries of each genotype were subjected to gross and microscopic analyses for each time point. Germ cell cysts, primordial, primary, and secondary follicles were defined as described [45]. We used antibodies against SOHLH1 [45], PLZF ( sc-22839, Santa Cruz, CA,USA), DDX4 (ab13840, Abcam, MA, USA), ATM phospho-S1981 (05-740, Millipore, CA, USA), and HORMAD1 proteins. Affinity purified, anti-HORMAD1 guinea pig and rabbit antibodies, were generated against part of HORMAD1 protein (amino acids 23-373) at Cocalico Biologicals (Lancaster, PA, USA).

### Meiotic chromosome analysis and immunostaining

Oocytes and spermatocytes for chromosome analyses were prepared essentially as described previously [62]–[63]. Ovaries and testes were incubated in trypsin-EDTA solution at 37°C for 15 min and washed briefly in PBS. Trypsinized ovaries and testes were pipetted repeatedly and centrifuged, followed by resuspension in PBS. Cell suspensions were placed on poly-L-lysine-coated slides containing 120 mM sucrose solution and 0.05% Triton X-100. The slides were fixed in 2% paraformaldehyde and 0.02% SDS for 1 hour at room temperature, washed in distilled water and air dried, and stored at −80°C before use. Immunostaining was preformed as described [62]–[63]. Slides were incubated with the primary antibody overnight at 4°C, washed with PBS and incubated with Alexa-488 and Alexa-595 (Invitrogen, CA, USA) secondary antibody (1∶300 dilutions) for 1 hour at room temperature. After washing with PBS, slides were mounted using VECTASHIELD medium with 4,6-diamidino-2-phenylindole (DAPI) (Vector Laboratories, CA, USA). SYCP2 rabbit and guinea pig immunoaffinity-purified antibody [48], DMC1 (sc-8973, Santa Cruz, CA, USA), RAD51 (ab1837, Abcam, CA, USA) and RPA (ab87272, Abcam, CA, USA) were used. Dr. Christer Höög (Karolinska Institutet, Sweden) kindly provided SYCP1 and SYCP3 rabbit immunoaffinity-purified antibodies. Dr. William R. Brinkley (Baylor College of Medicine, TX, USA) kindly provided anti-CREST human serum.

### Quantification of the immunofluorescence signal

Wild-type and mutant oocyte and spermatocyte spreads were stained at the same time with the same mixture of antibodies. In each experiment, when comparing wild type and mutants, imaging of the cells was performed on the same day with the same microscope and camera settings. PerkinElmer Volocity software 5.3 was used to control for possible changes in illumination during the course of imaging and measurement of the immunofluorescence. We measured total immunofluorescence of γH2AX in identical-sized rectangles that were placed over the cell boundaries. Fifty individual leptotene stage spreads from wild type, mutant spermatocytes and oocytes were subjected to immunofluorescence analysis. A two-tailed non-parametric Wilcoxon–Mann–Whitney two-sample rank-sum test was used for sample comparison.

### Electron microscopy analysis

Testes were dissected into ∼0.25 cm pieces and fixed in 1x PBS with 2% formaldehyde and 3% glutaraldehyde (Ted Pella Inc., CA, USA), for 2 hr at room temperature. Samples were treated with 0.5% uranyl acetate and osmium tetraoxide, dehydrated with ethanol, and embedded in LX-112 medium (Ladd Research Industries, VT, USA). The samples were polymerized in a 70°C oven for 2 days. Ultrathin sections (70 –100 nm) were cut in a Leica Ultracut microtome (Leica, IL, USA), stained for 5 min in 1% aqueous uranyl acetate and 2 min in 1% aqueous lead citrate at room temperature in a Leica EM Stainer, and examined by a JEM 1010 transmission electron microscope (JEOL, MA, USA) at an accelerating voltage of 80 kV. Digital images were obtained using AMT Imaging System (Advanced Microscopy Techniques Corp, MA, USA).

### Western blot analysis and phosphatase treatment

Western blot analysis was performed essentially as described previously [64]. Postnatal day 5, 8, 14, 18 and 21 testes were collected and lysed in RIPA buffer (50 mM Tris-HCl, pH 7.4, 1% NP-40, 0.25% sodium deoxycholate, 150 mM NaCl, 1 mM EDTA with or without protease inhibitor cocktail (Roche)). Total protein (20 µg) was loaded onto the SDS–PAGE gel, transferred to nitrocellulose membrane, and immunoblotted with HORMAD1 rabbit antibody (1∶5000 dilutions). Varying amounts of protein phosphatase I (Sigma P7937, MO, USA) were incubated with 20 µg of 18 day old wild-type total testes lysates, for 30 min on ice. Phosphatase treated testes lysates were electrophoresed, transferred to nitrocellulose membrane and immunoblotted with anti-HORMAD1 rabbit antibody.

### RNA isolation, RT-PCR, quantitative real-time PCR (Q-PCR), and microarray analysis

RT-PCR and quantitative real time PCR were performed as described previously [45], [65]. We used previously published oligonucleotide sequences corresponding to *Rnh2*, *Mov10l1*, *Pramel3, Usp26, Fthl17, Tex11*, *Tex13* [66], and *Hormad1 (*forward 5′ CTGCTGACACCAAGAAAGCA 3′- and reverse 5′- CCTGGTGGTTGGTAATCTGG -3′) and *Hormad2* (forward 5′ GCTCATCAGGGGCTAGACTG 3′- and reverse 5′- TGGTTCGCTGACCTTCTTCT -3′) primers. Q-PCR was performed using the SyberGreen PCR Master Mix (Bio-Red, CA, USA) and probe set specific for each gene under investigation. Each sample was analyzed in triplicate from at least three independent wild type and *Hormad1^−/−^* testes cDNA samples. The relative amount of transcripts was calculated by the ΔΔCT method as described by Applied Biosystems, and normalized to *β-actin*. The average and standard errors were calculated for the triplicate measurements, and the relative amount of target gene expression for each sample was plotted. Student's *t* test was used to compute the *P* values. Significance was defined as a *P* value<0.05.

RNA expression arrays on wild-type and *Hormad1^−/−^* 2 week old testes were performed on the Illumina BeadChip MouseWG-6 2.0 arrays and analyzed as previously described (GEO accession numbers, GSE21524) [67]–[68].

### Superovulation and oocyte collection in mouse

Mouse oocytes and pre-implantation embryos were collected by using standard protocols for timed mating [69]. Briefly, 4 to 6 weeks female mice were superovulated by injection of 5 IU of pregnant mare serum gonadotropin (PMSG) (Prospec, Rehovot, Israel) and 48 h later 5 IU of human chorionic gonadotropin (hCG) (Sigma, MO, USA). Females were placed individually with 10-week old males immediately after the injection of hCG. Collection of one cell, two cell, four cell, eight cell, and blastocyst stages was performed at 24 h, 42 h, 68 h, and 96 h after hCG injection, by flushing the oviducts and uterine horns with HEPES-buffered media under the microscope. Removal of the cumulus cells was achieved in HEPES-buffered media containing 1 mg/ml hyaluronidase (Sigma, MO, USA).

### FISH analysis

Oocytes and embryos were exposed to a hypotonic solution containing 0.1% sodium citrate, 0.1% bovine serum albumin for about 15 min and gently transferred onto the slides. Cells were fixed with fresh methanol: acetic acid (3∶1) solution dispensed carefully over the surface of the slide. The slides were air-dried and dehydrated at room temperature in a series of 70%, 85% and 100% ethanol solutions before FISH analysis.

Twenty BAC (bacterial artificial chromosomes) clones from a mouse RPCI-23 library representing sequences unique to specific chromosomes were selected from the UCSC Genome browser (http://genome.ucsc.edu) and NCBI (http://www.ncbi.nlm.nih.gov) databases (Table S1). Probe DNA extraction was performed according to the standard alkaline lysis method and labeled by standard nick translation with Spectrum Orange- or Spectrum Green-dUTP using a commercially available kit (Abbott/Vysis, IL, USA). Sequential fluorescence *in situ* hybridization experiments using a mixture of three probes were performed according to manufacturer's instructions (Abbott/Vysis, IL, USA). Each mixture contained a red, green and yellow (combined green/red) fluorescent probes. Probes were applied to slides, hybridized for 20 hr at 37°C, washed with 0.4×SSC/0.3% NP-40 for 2 minutes at 73°C and with 2×SSC/0.1% NP-40 for 1 minute at room temperature, and counterstained with DAPI. Digital FISH images were captured by a Power Macintosh G3 System using MacProbe software version 4.4 (Applied Imaging, CA, USA).

## Supporting Information

Figure S1 Generation of *Hormad1^−/−^* Mice. (A) Disruption of the *Hormad1* locus. The targeting construct and schematized genomic locus of *Hormad1* are shown. The neo cassette replaced HORMA domain encoding highly conserved exons 4 and 5, and introduced an Xba I restriction enzyme site into the locus. This Xba I site was used as a diagnostic for Southern blot analysis of ES cells electroporated with the targeting vector (data not shown). Arrows indicate genotyping primers used to distinguish wild-type and mutant alleles in the transgenic animals. (B) RT-PCR analysis of *Hormad1* knockout mice shows lack of transcript corresponding to *Hormad1* in the knockout animals. A faintly visible lower molecular weight band in the knockout (*−*/*−*), corresponds to a mutant transcript without exons 4 and 5. Removal of exons 4 and 5 causes a frameshift mutation. (C) Total protein was isolated from testes of 2 week old wild-type (+/+), *Hormad1^−/−^* (*−*/*−*) and *Hormad1* ^+/*−*^ (+/−) mice and Western blot analysis was performed with anti-HORMAD1 specific antibody. No significant amount of HORMAD1 was detected. The β-actin signal serves as a loading standard.
(0.18 MB TIF)

Figure S2 Adult testes are atrophied in *Hormad1^−/−^* Mice. (A) Reduced testicular size and weight in *Hormad1^−/−^* (KO) as compared to the wild-type (WT) testes from 4 week old male siblings. (B) Ratio of testes/body weight from wild-type (WT) and *Hormad1^−/−^* (KO) testes. Error bars represent the standard error of mean. Student's t test was used to calculate P values.
(0.18 MB TIF)

Figure S3 Excess apoptosis and increased apoptotic index in *Hormad1^−/−^* testes. (A-E) TUNEL assay in 1, 2, 3, 4, and 6 week old wild-type mouse testes. (F-J) TUNEL assay in 1, 2, 3, 4, and 6 week old *Hormad1^−/−^* mouse testis. (K) Apoptotic index was calculated in wild-type (WT) and *Hormad1^−/−^* mouse testis (KO) mice. Knockout testes had significantly higher apoptotic index at 2 weeks of post-natal life and beyond. Error bars represent the standard error of the mean. Error bars represent the standard error of the mean. Student's t test was used to calculate P values. P value was less than 0.001 at every time point except one week. Scale bar: 100 µM.
(3.02 MB TIF)

Figure S4 No germ cell loss in the *Hormad1^−/−^* ovary. At post-natal day 8, primordial, primary, and secondary follicles were detected in wild-type (A) and the *Hormad1^−/−^* ovaries (B). Anti-Lhx8 antibody was used to detect germ cells (brown stain). Histogram represents primordial follicles (PF), primary follicles (PrF), and a total number of secondary follicles (SF) in the wild-type (WT) and mutants (KO) (C). Every fifth section of wild-type (n = 6) and *Hormad1^−/−^* ovaries (n = 5) were counted. Error bars represent the standard error of the mean. Fisher's exact *t* test was used to calculate *P* values. *P* value between mutant and wild-type primordial follicles, primary, and secondary follicles was >0.5, and therefore not statistically significant. Bars, 50 µm.
(1.85 MB TIF)

Table S1 BAC clones used in FISH experiments.
(0.03 MB DOC)
