# Supplementary figures and images for: Hormad1 Mutation Disrupts Synaptonemal Complex Formation, Recombination, and Chromosome Segregation in Mammalian Meiosis
Source: PLoS Genet. 2010 Nov 4;6(11):e1001190. doi: 10.1371/journal.pgen.1001190 (PMC2973818; doi:10.1371/journal.pgen.1001190)

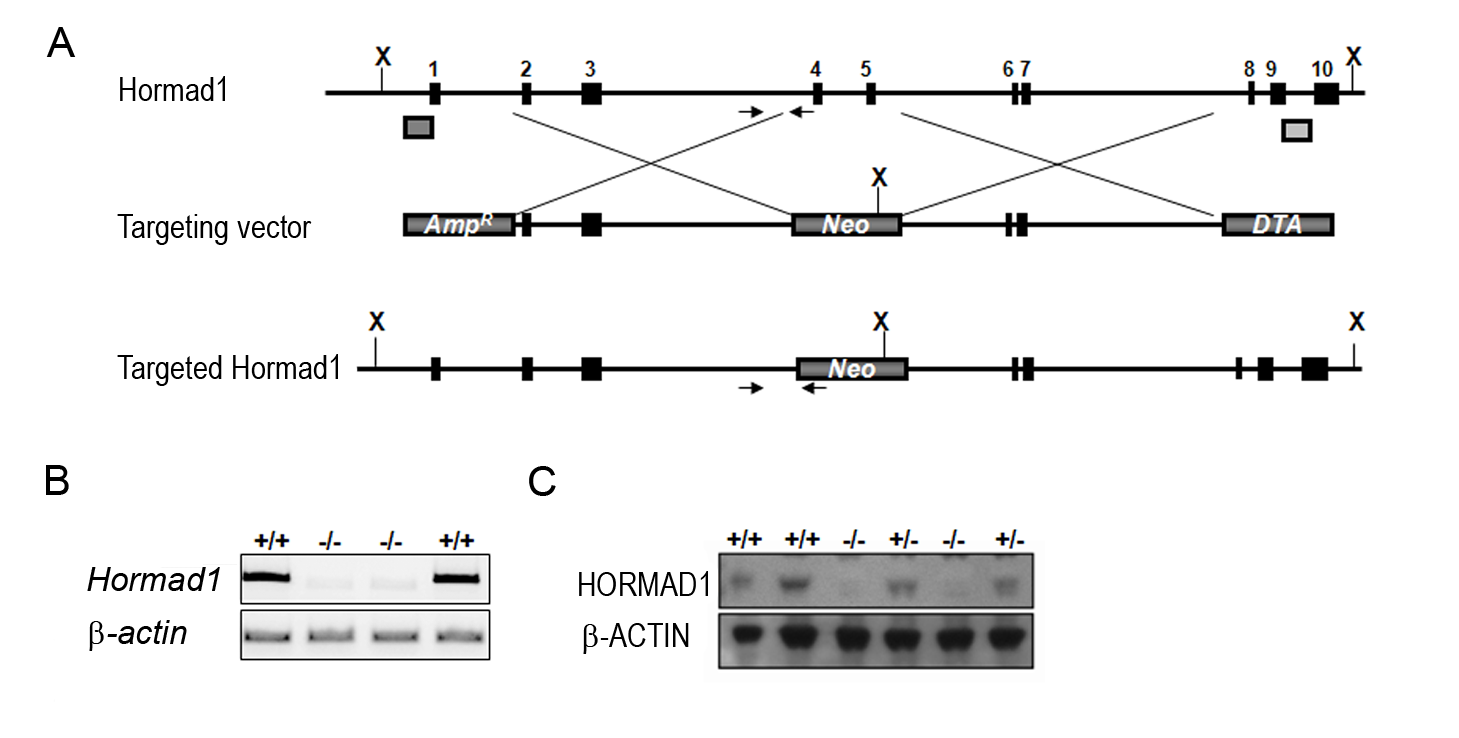

Supplement: Figure S1 — Generation of Hormad1−/− Mice. (A) Disruption of the Hormad1 locus. The targeting construct and schematized genomic locus of Hormad1 are shown. The neo cassette replaced HORMA domain encoding highly conserved exons 4 and 5, and introduced an Xba I restriction enzyme site into the locus. This Xba I site was used as a diagnostic for Southern blot analysis of ES cells electroporated with the targeting vector (data not shown). Arrows indicate genotyping primers used to distinguish wild-type and mutant alleles in the transgenic animals. (B) RT-PCR analysis of Hormad1 knockout mice shows lack of transcript corresponding to Hormad1 in the knockout animals. A faintly visible lower molecular weight band in the knockout (−/−), corresponds to a mutant transcript without exons 4 and 5. Removal of exons 4 and 5 causes a frameshift mutation. (C) Total protein was isolated from testes of 2 week old wild-type (+/+), Hormad1−/− (−/−) and Hormad1 +/− (+/−) mice and Western blot analysis was performed with anti-HORMAD1 specific antibody. No significant amount of HORMAD1 was detected. The β-actin signal serves as a loading standard. (0.18 MB TIF) [file pgen.1001190.s001.tif]

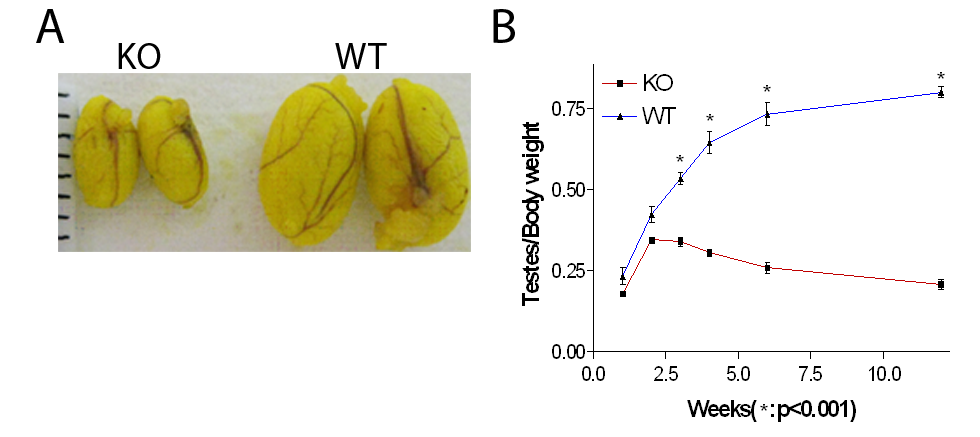

Supplement: Figure S2 — Adult testes are atrophied in Hormad1−/− Mice. (A) Reduced testicular size and weight in Hormad1−/− (KO) as compared to the wild-type (WT) testes from 4 week old male siblings. (B) Ratio of testes/body weight from wild-type (WT) and Hormad1−/− (KO) testes. Error bars represent the standard error of mean. Student's t test was used to calculate P values. (0.18 MB TIF) [file pgen.1001190.s002.tif]

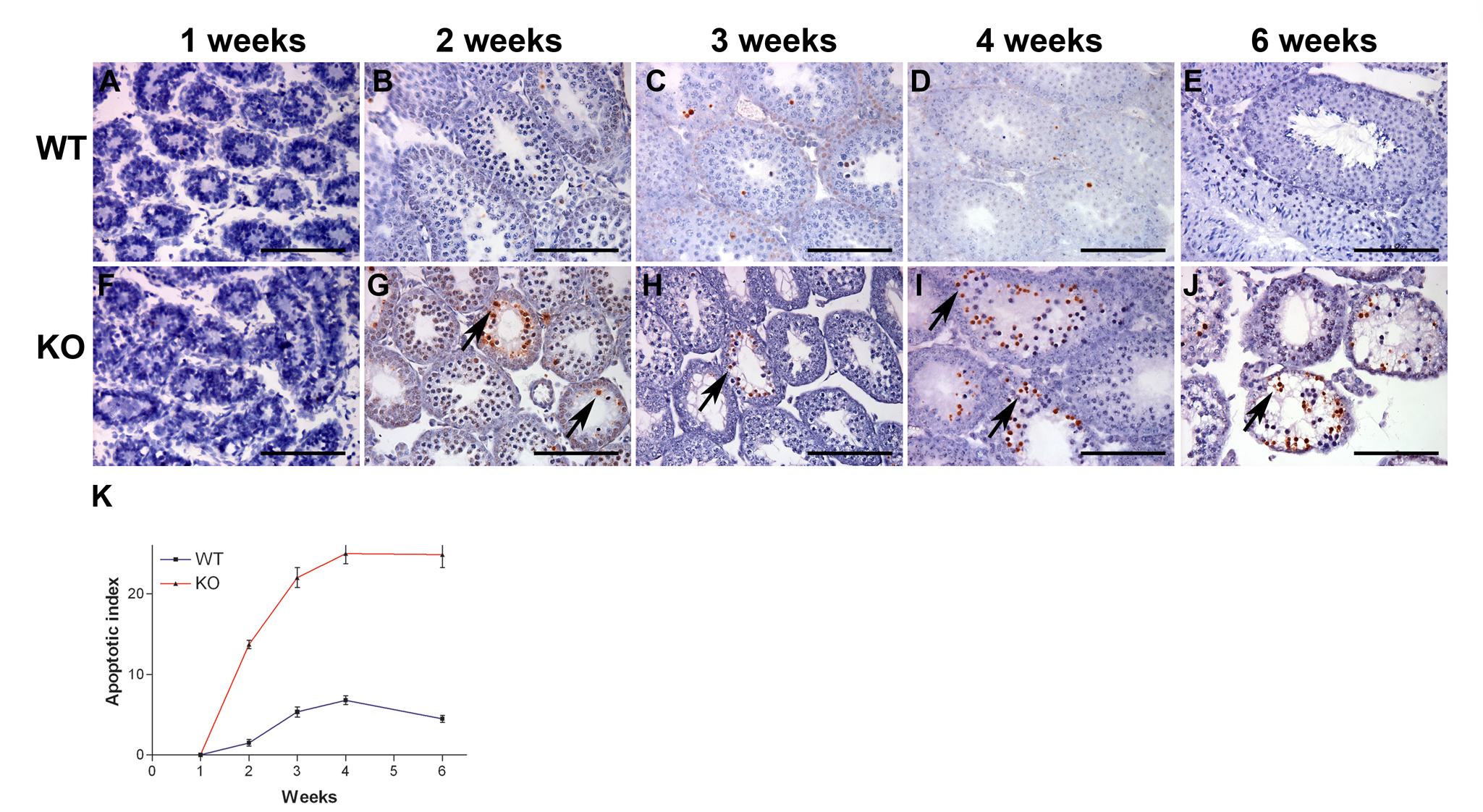

Supplement: Figure S3 — Excess apoptosis and increased apoptotic index in Hormad1−/− testes. (A-E) TUNEL assay in 1, 2, 3, 4, and 6 week old wild-type mouse testes. (F-J) TUNEL assay in 1, 2, 3, 4, and 6 week old Hormad1−/− mouse testis. (K) Apoptotic index was calculated in wild-type (WT) and Hormad1−/− mouse testis (KO) mice. Knockout testes had significantly higher apoptotic index at 2 weeks of post-natal life and beyond. Error bars represent the standard error of the mean. Error bars represent the standard error of the mean. Student's t test was used to calculate P values. P value was less than 0.001 at every time point except one week. Scale bar: 100 µM. (3.02 MB TIF) [file pgen.1001190.s003.tif]

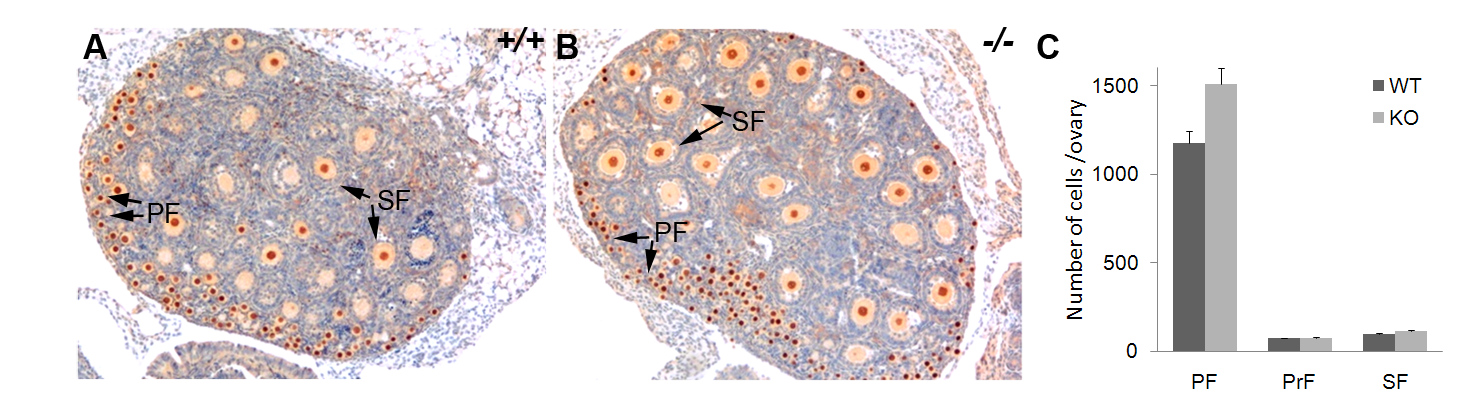

Supplement: Figure S4 — No germ cell loss in the Hormad1−/− ovary. At post-natal day 8, primordial, primary, and secondary follicles were detected in wild-type (A) and the Hormad1−/− ovaries (B). Anti-Lhx8 antibody was used to detect germ cells (brown stain). Histogram represents primordial follicles (PF), primary follicles (PrF), and a total number of secondary follicles (SF) in the wild-type (WT) and mutants (KO) (C). Every fifth section of wild-type (n = 6) and Hormad1−/− ovaries (n = 5) were counted. Error bars represent the standard error of the mean. Fisher's exact t test was used to calculate P values. P value between mutant and wild-type primordial follicles, primary, and secondary follicles was >0.5, and therefore not statistically significant. Bars, 50 µm. (1.85 MB TIF) [file pgen.1001190.s004.tif]
