# Supplementary material for: Hormad1 Mutation Disrupts Synaptonemal Complex Formation, Recombination, and Chromosome Segregation in Mammalian Meiosis
Source: PLoS Genet. 2010 Nov 4;6(11):e1001190. doi: 10.1371/journal.pgen.1001190 (PMC2973818; doi:10.1371/journal.pgen.1001190)
Supplement: Table S1 — BAC clones used in FISH experiments. (0.03 MB DOC) [file pgen.1001190.s005.doc]

| Chromosome location | BAC clone |
| --- | --- |
| 2qC1.3 | RP23-277I22 |
| 6qC1 | RP23-277H8 |
| 11qA1 | RP23-277K5 |
| 18qB1 | RP23-277H20 |
| 19qA | RP23-238G6 |
| XqA1.1 | RP23-237A1 |
